# Maturation and degeneration of the human brainstem across the adult lifespan

**DOI:** 10.18632/aging.203183

**Published:** 2021-06-11

**Authors:** Mustapha Bouhrara, Luis E. Cortina, Nikkita Khattar, Abinand C. Rejimon, Samuel Ajamu, Defne S. Cezayirli, Richard G. Spencer

**Affiliations:** 1 Laboratory of Clinical Investigation, National Institute on Aging, National Institutes of Health, Baltimore, MD 21224, USA; 2 Laboratory of Cardiovascular Science, National Institute on Aging, National Institutes of Health, Baltimore, MD 21224, USA

**Keywords:** brainstem, myelin water fraction, diffusion tensor imaging, relaxation rates, aging

## Abstract

Brainstem tissue microstructural properties change across the adult lifespan. However, studies elucidating the biological processes that govern brainstem maturation and degeneration *in-vivo* are lacking. In the present work, conducted on a large cohort of 140 cognitively unimpaired subjects spanning a wide age range of 21 to 94 years, we implemented a multi-parameter approach to characterize the sex- and age differences. In addition, we examined regional correlations between myelin water fraction (MWF), a direct measure of myelin content, and diffusion tensor imaging indices, and transverse and longitudinal relaxation rates to evaluate whether these metrics provide information complementary to MWF. We observed region-dependent differences in myelin content and axonal density with age and found that both exhibit an inverted U-shape association with age in several brainstem substructures. We emphasize that the microstructural differences captured by our distinct MRI metrics, along with their weak associations with MWF, strongly indicate the potential of using these outcome measures in a multi-parametric approach. Furthermore, our results support the gain-predicts-loss hypothesis of tissue maturation and degeneration in the brainstem. Indeed, our results indicate that myelination follows a temporally symmetric time course across the adult life span, while axons appear to degenerate significantly more rapidly than they mature.

## INTRODUCTION

Postmortem histological studies have identified age-related changes in the brainstem, including volumetric midbrain atrophy [1, 2] and neuronal loss [3] that may account for the neurofunctional decline observed in the elderly. Just as important, multiple studies have also suggested early brainstem involvement in the prodromal stage of Alzheimer’s Disease (AD) and Parkinson’s Disease (PD) [4–7]. In addition, particular brainstem nuclei that undergo specific types of degeneration have served as potential targets of surgical interventions such as deep brain stimulation in PD [8]. Despite the brainstem’s involvement in neurodegeneration and aging and representing a therapeutic target for surgical intervention, most of our knowledge of the brainstem microstructure is derived from *ex-vivo* and postmortem studies. Although these investigations have provided critical insights into our understanding of the brainstem, they cannot be performed in real-time on living subjects with limited ability to perform correlative studies with cognitive performance and treatment. Therefore, characterizing age-related differences *in vivo* is essential for identifying biomarkers of tissue microstructure, distinguishing age-dependent changes from neurodegeneration, and evaluating therapeutic interventions.

The complex anatomical structure of the brainstem and the relative lack of tissue contrast render MRI-based studies difficult [9]. High-spatial resolution imaging is critical to minimize partial volume effects; this can also increase tissue contrast between gray matter (GM) and white matter (WM). In pioneering work, Lambert and colleagues identified widespread alterations in brainstem substructures and established a baseline of microstructural changes with age using multiple quantitative MRI measures [10]. Specifically, they reported linear associations between apparent transverse and longitudinal relaxation rates (*R_1_*, R_2_*), magnetization transfer saturation (MTS), and proton density with age in a number of brainstem regions. They attributed this to axonal loss or demyelination in the WM, with GM changes being secondary to iron deposition. While these processes could account for their observations, conventional quantitative MRI measures such as those employed in that study are also sensitive to several other tissue properties and model-based assumptions [11–14]. Furthermore, while Lambert and colleagues provided critical insights into microstructural age-related changes, their results are inconsistent with our previous demonstration of a nonlinear association between myelin content and age in several brainstem substructures [15]. This is likely due to smaller sample size in Lambert et al., especially in the age range of 35 and 65 years.

To address these limitations, we studied a larger cohort, with an improved age distribution, of cognitively unimpaired subjects (*N* = 140) across the extended age range of 21-94 years. Our main objectives are: First, building on Lambert et al. and our previous work [10, 15], we sought to characterize age-and sex-related microstructural correlates by determining the diffusion tensor imaging (DTI) indices of fractional anisotropy (FA) and mean, radial, and axial diffusivities (MD, RD, and AxD), as well as longitudinal and transverse relaxation rates (*R_1_*, *R_2_*), and myelin water fraction (MWF) quantification using our recently-introduced method [16–21], within selected brainstem substructures. Second, we compared MWF estimates with widely used markers of myelin content (RD, *R_1_*, *R_2_*), axonal damage (AxD), and tissue composition (FA, MD) to determine whether the information provided by these biomarkers is complimentary or is redundant to MWF. We further evaluate whether a reduction in myelin content, as measured by MWF, could explain the age-related changes seen in some of these parameters, which are much less specific for myelin content. Indeed, relaxation rate, and DTI index values cannot readily be attributed to any particular microstructural parenchymal features, since they are susceptible to multiple tissue characteristics and processes, including axonal damage or loss, geometry of crossing fibers, myelin content, iron content, local cellular infiltration and proliferation, and edema [12, 22–24]. Third, we examined the gain-predicts-loss hypothesis of tissue maturation and degeneration in the brainstem using these MR parameters. Lastly, we sought to provide reference values for all these parameters in the brainstem in normative aging.

## RESULTS

### Age and sex effects on MWF, relaxation rates, and DTI indices

**Figure 1:**
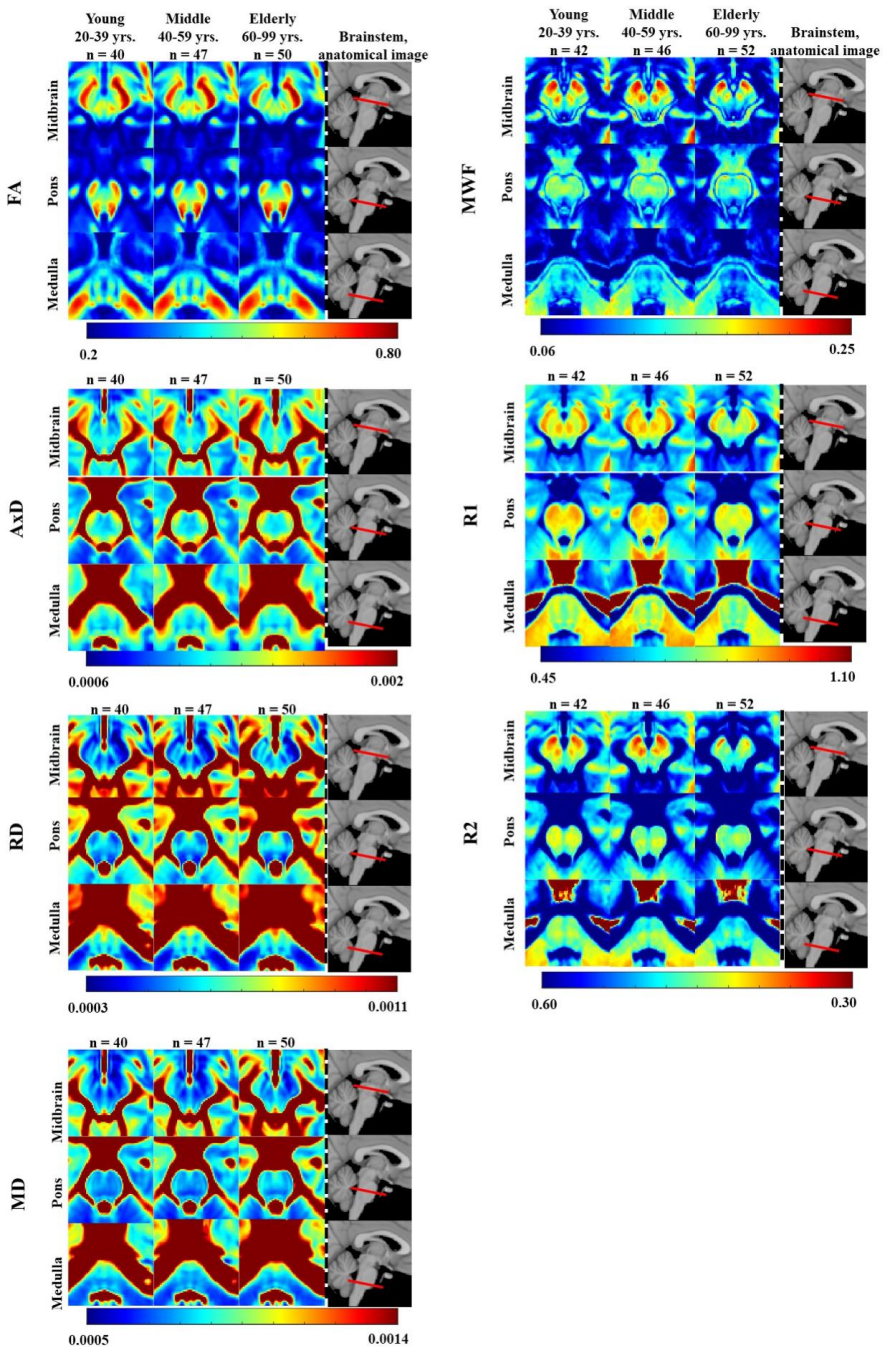
**FA, AxD, RD, MD, MWF, *R_1_*, and *R_2_* represented as averaged participant maps calculated for three age groups.** Three representative slices covering respectively the midbrain, pons, and medulla are displayed. The red bars on the anatomical images indicate the location of these slices. Visual inspection indicates an increase in *R_1_*, *R_2_*, and MWF from early adulthood, 20-29 years, through middle age, followed by a decrease in several brainstem substructures, and a more generally monotonic decrease in FA. Inspection of AxD, RD, and MD demonstrated a slight decrease from early adulthood through middle age followed by an increase in several brainstem substructures.

Figure 1 illustrates the regional differences in MWF, *R_1_*, *R_2_*, FA, MD, AxD, and RD across the adult lifespan, represented with brain parameter maps averaged from participants within three different age intervals of our cohort: young (21-39 years), middle-aged (40-59 years), and elderly (60-94 years). Three representative slices were displayed covering the main anatomical subdivisions of the brainstem, that is, midbrain, pons, and medulla. As expected, the parameter maps exhibit tissue contrast between different brainstem substructures and across age intervals. Visual inspection suggests increases in MWF, *R_1_*, and *R_2_* values and decreases in MD, AxD, and RD values from early adulthood, 20-39 years, until middle age, 40-59 years, followed by decreases in MWF, *R_1_*, and *R_2_* values and increases in MD, AxD, and RD values at older ages, within several brainstem regions. In contrast, visual inspection of FA maps suggests overall decreases with age within most brainstem regions. Furthermore, we note that the superior brainstem regions, especially the midbrain, exhibit greater MWF, *R_1_*, *R_2_*, and FA values and lower RD, MD, and AxD values in comparison to the inferior brainstem substructures such as the pons and medulla (Supplementary Tables 1–7).

**Figure 2:**
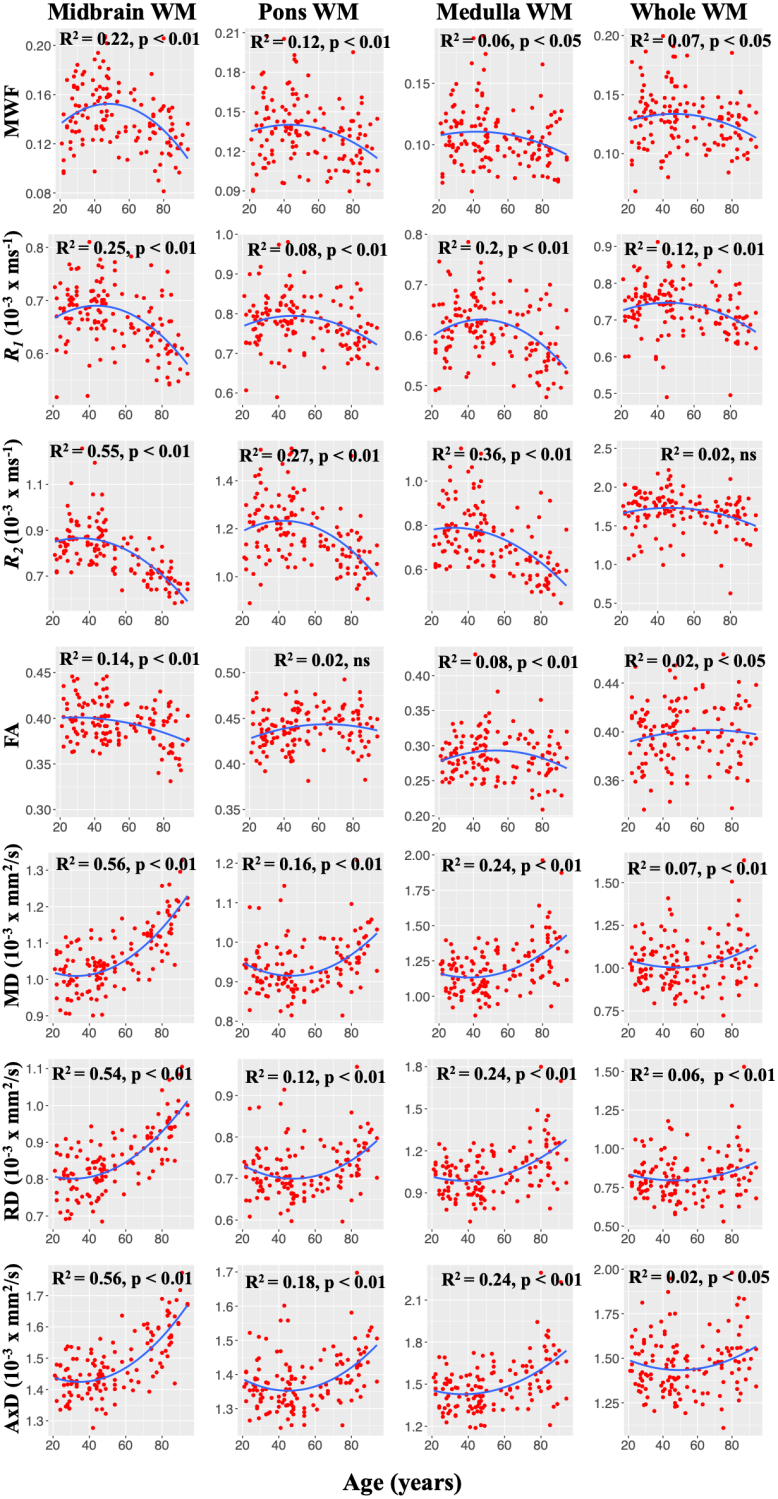
**Representative plots of MWF, *R_1_*, and *R_2_*, and DTI indices values as a function of age.** Note that, unlike FA, MWF, *R_1_*, *R_2_*, MD, RD, and AxD conform to nonlinear regional trends with age. For each ROI, the coefficient of determination, *R*^2^, and the significance of the linear regression model, *p*, are reported.

Figure 2 illustrates the representative plots of derived MWF, relaxation rates, and DTI indices values from all subjects as a function of age for midbrain, pons, medulla, and whole brainstem WM. Visual inspection of the plots suggest an increase of MWF, *R_1_*, *R_2_,* until middle age followed by a decrease in all ROIs examined, in agreement with Figure 1. Additionally, the best-fit curves of MD, RD, and AxD all demonstrated nonlinear associations with age (Figure 2). In contrast, FA indicated linear regional associations with age. Our linear regression analysis showed significant (*p* < 0.05) associations with age after FDR correction in several brainstem subdivisions (Table 1). Lastly, the linear regression analysis also showed a significant or close to significant sex effect on these MR parameters in various regions examined (Table 1). In these ROIs, women showed a more rapid decline of MWF as compared to men as well as higher FA, AxD, and MD values in comparison to males (Table 1). Additional plots representing each ROI investigated can be found in our supplementary material (Supplementary Figures 1–7).

**Table 1:** Significance of sex, age, and age^2^ terms incorporated in the linear regression.

	***MWF***			***R_1_***			***R_2_***			**FA**			**MD**			**RD**			**AxD**			
	**Sex**	**Age**	**Age^2^**	**Sex**	**Age**	**Age^2^**	**Sex**	**Age**	**Age^2^**	**Sex**	**Age**	**Age^2^**	**Sex**	**Age**	**Age^2^**	**Sex**	**Age**	**Age^2^**	**Sex**	**Age**	**Age^2^**	**Number of voxels**
	***p***	***p***	***p***	***p***	***p***	***p***	***p***	***p***	***p***	***p***	***p***	***p***	***p***	***p***	***p***	***p***	***p***	***p***	***p***	***p***	***p***	
***Superior cerebellar***	> 0.1	**<0.01**	> 0.1	> 0.1	**<0.05**	> 0.1	> 0.1	**<0.01**	> 0.1	> 0.1	**<0.01**	> 0.1	**<0.1**	> 0.1	> 0.1	**<0.1**	**<0.01**	> 0.1	**<0.01**	**<0.01**	> 0.1	250
***Middle cerebellar***	> 0.1	> 0.1	> 0.1	> 0.1	> 0.1	> 0.1	> 0.1	**<0.01**	> 0.1	> 0.1	> 0.1	> 0.1	> 0.1	> 0.1	> 0.1	> 0.1	> 0.1	> 0.1	> 0.1	> 0.1	> 0.1	1934
***Inferior cerebellar***	> 0.1	**<0.01**	> 0.1	> 0.1	**<0.05**	> 0.1	> 0.1	**<0.01**	**<0.1**	**<0.1**	> 0.1	> 0.1	> 0.1	> 0.1	**<0.1**	> 0.1	> 0.1	**<0.1**	> 0.1	**<0.01**	**<0.1**	253
***Cerebral peduncle***	> 0.1	> 0.1	**<0.05**	> 0.1	> 0.1	**<0.05**	> 0.1	**<0.01**	**<0.1**	**<0.1**	**<0.01**	> 0.1	> 0.1	**<0.01**	> 0.1	> 0.1	**<0.01**	> 0.1	> 0.1	<0.1	> 0.1	2549
***Corticospinal***	> 0.1	**<0.05**	> 0.1	> 0.1	<0.1	> 0.1	> 0.1	**<0.01**	> 0.1	> 0.1	> 0.1	> 0.1	> 0.1	> 0.1	> 0.1	> 0.1	> 0.1	> 0.1	> 0.1	> 0.1	**<0.1**	1142
***Pontine***	> 0.1	**<0.05**	**<0.01**	> 0.1	> 0.1	> 0.1	> 0.1	**<0.01**	**<0.05**	> 0.1	> 0.1	> 0.1	> 0.1	**<0.01**	**<0.05**	> 0.1	> 0.1	**<0.1**	> 0.1	**<0.01**	**<0.01**	481
***Lemniscus***	> 0.1	**<0.05**	> 0.1	> 0.1	> 0.1	> 0.1	> 0.1	**<0.01**	**<0.01**	> 0.1	**<0.05**	> 0.1	**<0.05**	**<0.05**	**<0.1**	> 0.1	> 0.1	> 0.1	**<0.05**	**<0.01**	> 0.1	418
***Whole WM***	> 0.1	<0.1	> 0.1	> 0.1	**<0.05**	> 0.1	> 0.1	> 0.1	> 0.1	> 0.1	> 0.1	> 0.1	**<0.1**	**<0.05**	> 0.1	**<0.1**	**<0.05**	> 0.1	**<0.1**	**<0.05**	**<0.01**	28977
***Midbrain WM***	> 0.1	**<0.01**	**<0.01**	> 0.1	**<0.01**	**<0.01**	> 0.1	<0.01	**<0.01**	> 0.1	**<0.01**	**<0.1**	**<0.1**	**<0.01**	**<0.01**	> 0.1	**<0.01**	**<0.01**	**<0.05**	**<0.01**	**<0.01**	8992
***Pons WM***	> 0.1	**<0.05**	> 0.1	> 0.1	**<0.01**	**<0.05**	> 0.1	<0.01	**<0.01**	> 0.1	> 0.1	> 0.1	> 0.1	**<0.01**	**<0.01**	> 0.1	**<0.01**	**<0.01**	> 0.1	**<0.01**	**<0.01**	11577
***Medulla WM***	> 0.1	**<0.05**	> 0.1	> 0.1	**<0.01**	**<0.01**	> 0.1	<0.01	**<0.05**	**<0.05**	> 0.1	> 0.1	**<0.1**	**<0.01**	**<0.05**	**<0.1**	**<0.01**	**<0.05**	> 0.1	**<0.01**	**<0.05**	1838
***Red nucleus***	> 0.1	**<0.01**	**<0.01**	> 0.1	**<0.01**	**<0.05**	> 0.1	<0.01	**<0.05**	> 0.1	> 0.1	**<0.05**	> 0.1	**<0.01**	**<0.01**	> 0.1	**<0.05**	**<0.01**	> 0.1	**<0.01**	**<0.05**	646
***Subthalamic***	> 0.1	**<0.01**	**<0.01**	<0.1	> 0.1	**<0.05**	> 0.1	> 0.1	> 0.1	> 0.1	> 0.1	> 0.1	> 0.1	**<0.01**	**<0.01**	> 0.1	**<0.01**	**<0.01**	> 0.1	**<0.01**	**<0.01**	280
***Substantia nigra***	> 0.1	**<0.05**	**<0.05**	> 0.1	> 0.1	**<0.05**	> 0.1	<0.01	**<0.05**	> 0.1	**<0.01**	> 0.1	> 0.1	**<0.01**	**<0.05**	> 0.1	**<0.01**	**<0.05**	> 0.1	<0.1	**<0.05**	537

Bold indicates significance (p < 0.05). All p-values presented are obtained after the FDR correction.

This Table shows the results derived from MWF, R1, R2, FA, MD, RD, and AxD.

### Regional correlations between MWF and relaxation rates or DTI indices

**Figure 3:**
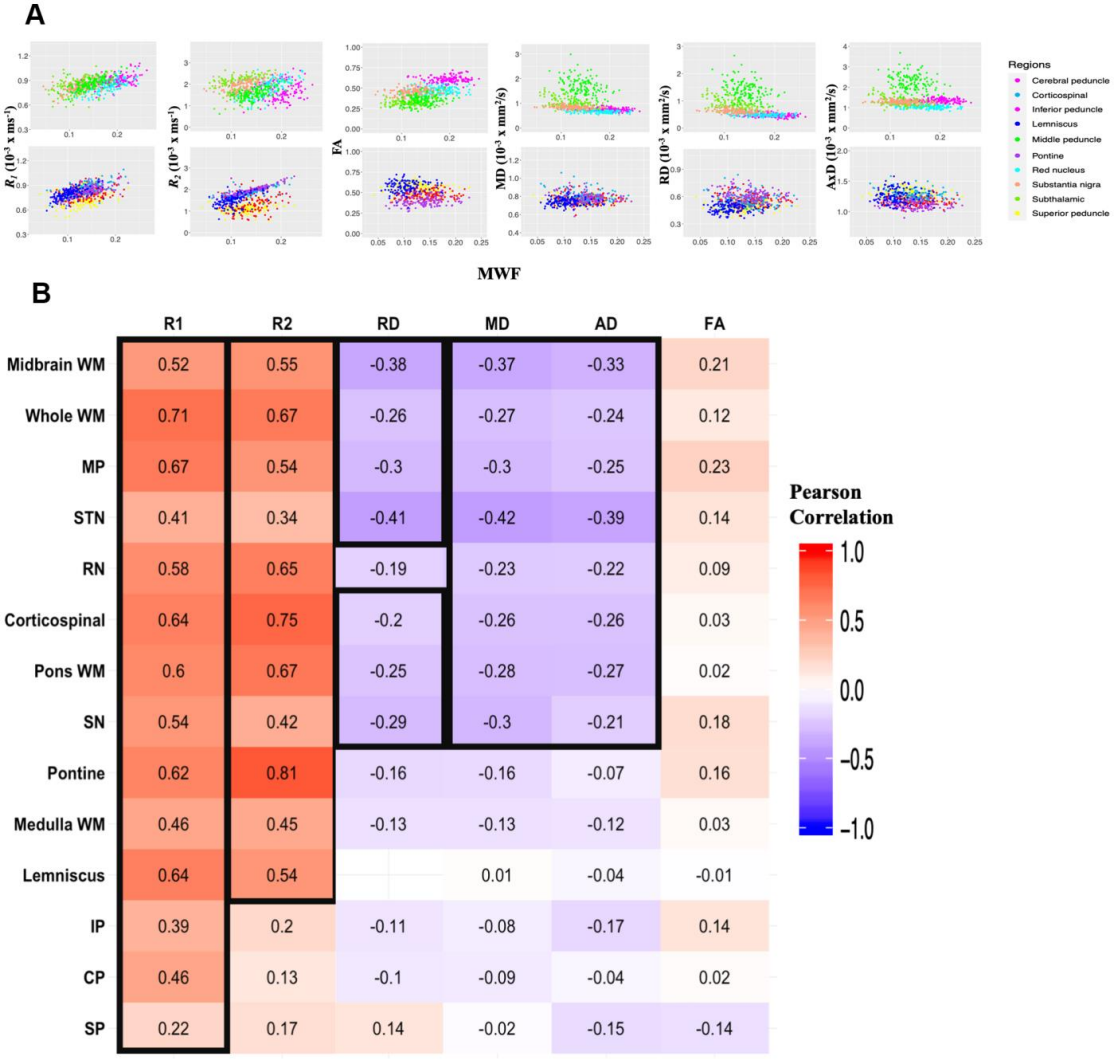
**Regional correlations between MWF and relaxation rates or DTI indices.** (**A**) Scatter plots illustrating the association between quantitative MRI measures and MWF across the 10 independent ROIs. Plots were split into 5 ROIs each for optimal visualization of the correlation trends between parameters. ROIs are indicated by different colors, matching those of Figure 5. Each participant is represented by a single dot. Pearson correlation analysis was conducted with results shown in Table 3. *R*_1_, *R*_2_ and FA exhibit a positive correlation with MWF while MD, RD, and AD are negatively correlated with MWF. (**B**) Correlational matrix providing the linear correlation coefficients values of each parameters against MWF for each ROI. Cell values in a black box represent Pearson correlation coefficients that were statistically significant after FDR (*p* < 0.05). The ROIs are ranked in order of decreasing mean *R*^2^ values across all metrics. Similarly, the parameters themselves are ranked in descending order of mean *R*^2^ values across all ROIs. MP, middle cerebellar peduncle; STN, subthalamic nucleus; RN, red nucleus; SN, substantia nigra; IP, inferior cerebellar peduncle; CP, cerebral peduncle; SP, superior cerebellar peduncle.

Correlations between relaxation rates or DTI indices and MWF are shown in Figure 3. Pearson correlations of the combined data across 10 independent ROIs studied and over all participants demonstrate significant correlations (*p* < 0.01) that remained statistically significant after FDR. Specifically, *R_1_ vs*. MWF, *R_2_ vs*. MWF, and FA *vs*. MWF exhibited significant positive correlations while RD *vs.* MWF, MD *vs*. MWF, and AxD *vs.* MWF showed significant negative correlations (Figure 3A). However, in terms of effect size, only the relationships between *R_1_* or *R_2_* and MWF exhibited a Pearson correlation coefficient above 0.3, with all other parameters exhibiting weak or very weak correlation with MWF. Finally, Figure 3B illustrates the correlation coefficient value for each ROI and for each parameter against MWF. Overall, *R_1_*, and to a lesser extent, *R_2_*, showed moderate-to-strong regional correlations with MWF as compared to all other metrics which exhibited modest correlations with MWF.

**Table 2:** The slopes and standard error (SE) of the maturation and degeneration phases of the standardized MWF, R1, R2, and DTI indices for six brainstem substructures.

	**Maturation and degeneration slopes ± SE**								
		**Slope of** **maturation**	**Slope of degeneration**	***p-value***			**Slope of** **maturation**	**Slope of degeneration**	***p-value***
**MWF**	***Cerebral peduncle***	0.039±0.006	-0.024±0.005	> 0.1	**MD**	***Cerebral peduncle***	-0.125±0.006	-0.016±0.004	NA
	***Pontine***	0.022±0.007	-0.021±0.004	> 0.1		***Pontine***	0.006±0.005	-0.050±0.006	**<0.01**
	***Midbrain WM***	0.038±0.006	-0.035±0.005	> 0.1		***Midbrain WM***	-0.003±0.004	-0.048±0.004	NA
	***Red nucleus***	0.027±0.005	-0.036±0.005	> 0.1		***Red nucleus***	0.008±0.005	-0.074±0.006	**<0.01**
	***Subthalamic nucleus***	0.031±0.006	-0.034±0.005	> 0.1		***Subthalamic nucleus***	0.023±0.004	-0.042±0.004	**<0.01**
	***Substantia nigra***	0.019±0.006	-0.027±0.004	> 0.1		***Substantia nigra***	0.002±0.005	-0.064±0.006	**<0.01**
***R_1_***	***Cerebral peduncle***	0.017±0.005	-0.029±0.005	> 0.1	**RD**	***Cerebral peduncle***	-0.234±0.008	-0.018±0.004	NA
	***Pontine***	0.013±0.006	-0.015±0.005	> 0.1		***Pontine***	0.006±0.005	-0.048±0.007	**<0.01**
	***Midbrain WM***	0.009±0.009	-0.007±0.004	> 0.1		***Midbrain WM***	0.014±0.009	-0.034±0.003	**<0.05**
	***Red nucleus***	0.006±0.005	-0.042±0.005	**<0.01**		***Red nucleus***	0.017±0.005	-0.061±0.006	**<0.01**
	***Subthalamic nucleus***	0.024 ±0.003	-0.020±0.002	> 0.1		***Subthalamic nucleus***	0.000±0.003	-0.046±0.004	**<0.01**
	***Substantia nigra***	0.013±0.005	-0.022±0.005	> 0.1		***Substantia nigra***	0.001±0.004	-0.030±0.005	**<0.01**
***R_2_***	***Cerebral peduncle***	0.039±0.012	-0.020±0.003	> 0.1	**AxD**	***Cerebral peduncle***	0.007±0.007	-0.013±0.005	> 0.1
	***Pontine***	0.020±0.007	-0.025±0.005	> 0.1		***Pontine***	0.009±0.005	-0.030±0.005	**<0.01**
	***Midbrain WM***	0.005±0.005	-0.016±0.006	> 0.1		***Midbrain WM***	-0.003±0.004	-0.052±0.004	NA
	***Red nucleus***	0.003±0.003	-0.033±0.004	**<0.01**		***Red nucleus***	0.003±0.005	-0.028±0.005	**<0.01**
	***Subthalamic nucleus***	0.163±0.006	-0.003±0.002	**<0.01**		***Subthalamic nucleus***	0.034±0.004	-0.041±0.004	> 0.1
	***Substantia nigra***	0.023±0.005	-0.011±0.004	> 0.1		***Substantia nigra***	0.008±0.005	-0.074±0.006	**<0.01**

Bold indicates a significant (p < 0.05) difference between the rate of maturation and the rate of degeneration.

### Rates of brainstem tissue maturation and degeneration

**Figure 4:**
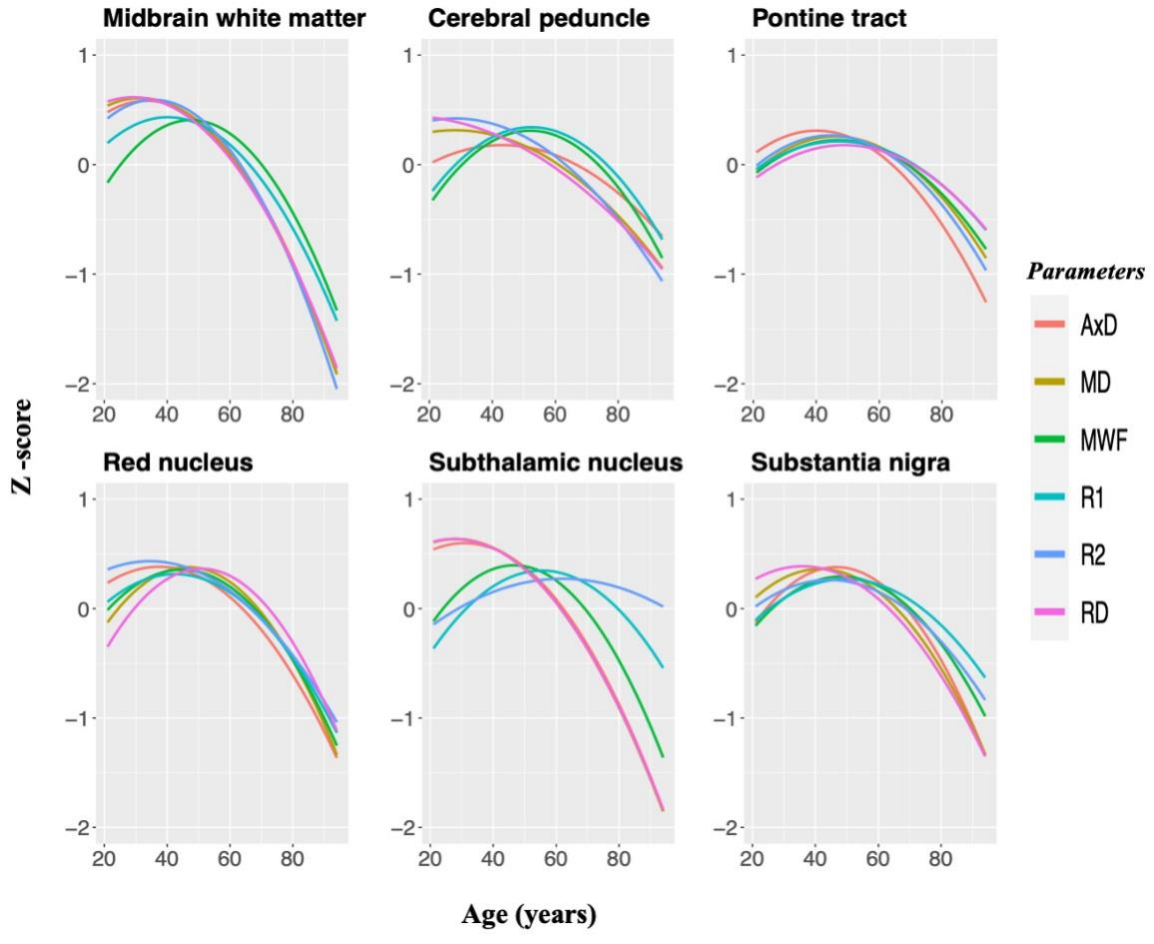
**MWF, relaxation times, and DTI indices standardized and plotted as a function of age for six brainstem substructures to illustrate similarities and differences between MR metrics in the brainstem microstructural maturation and degeneration across the adult lifespan.** Three white matter regions and three gray matter nuclei were chosen specifically since they demonstrated significant quadratic associations with age across all of these parameters. Diffusivity indices were inverted for easier comparisons.

Figure 4 provides a comparison of brainstem maturation with respect to our MRI outcome measures for several substructures. As seen, although all parameters evaluated demonstrated nonlinear associations with age, the midbrain, the cerebral peduncle, and the subthalamic nucleus exhibit distinct associations between MWF and *R_1_* or DTI indices and *R_2_* over the lifespan. Specifically, in these substructures, DTI indices and *R_2_* followed a similar trend with age and peaked at an earlier age as compared to MWF and *R_1_* trajectories; this is consistent with the correlation analysis results indicating strong correlation between MWF and *R_1_* (Figure 3). We also note that the lifespan associations with age observed in the red nucleus, the pontine tract, and the substantia nigra did not differ across any of these parameters.

**Figure 5:**
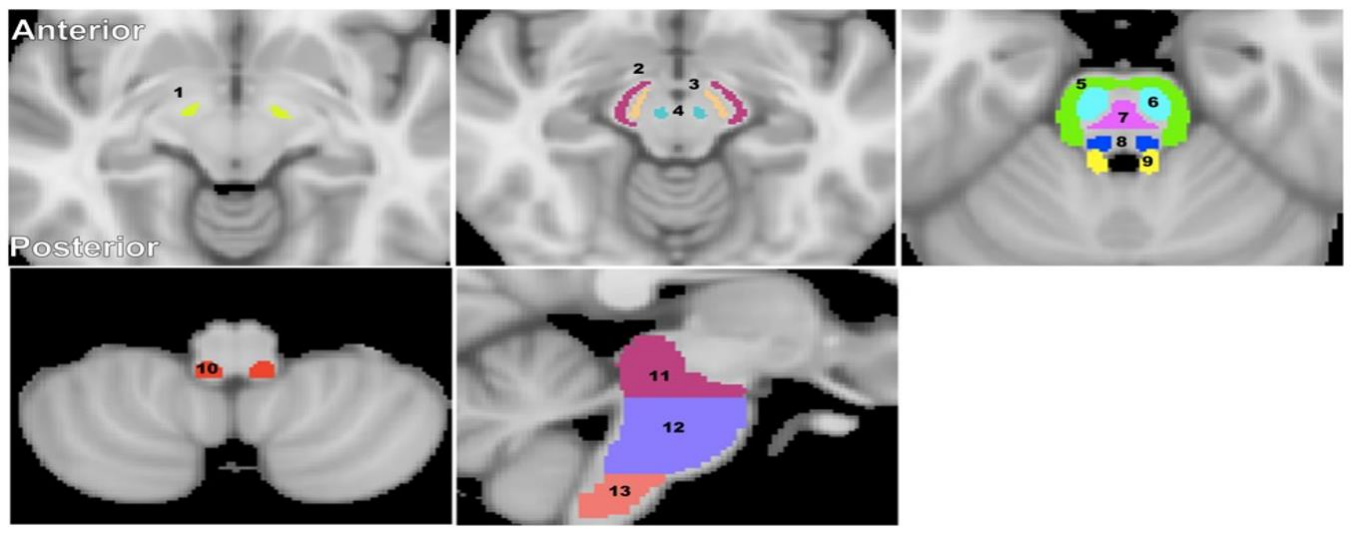
**Visualization of the brainstem WM and GM ROIs studied.** (**1**) Subthalamic nucleus, (**2**) Cerebral peduncle, (**3**) Substantia nigra, (**4**) Red nucleus, (**5**) Middle cerebellar peduncle, (**6**) Corticospinal tract, (**7**) Pontine tract, (**8**) Lemniscus tract, (**9**) Superior cerebellar peduncle, (**10**) Inferior cerebellar peduncle, (**11**) Midbrain, (**12**) Pons, and (**13**) Medulla. The brainstem structural images were obtained from the standard MNI atlas. Representative slices were chosen for visualization.

Table 2 provides values for the rates of maturation and degeneration for each ROI and for each MR parameter evaluated. We found that the rates of maturation and degeneration in MWF are similar across all substructures examined, with differences between them not reaching statistical significance. The absolute magnitudes of the maturation and degeneration rates differed for the diffusivity indices in most substructures as well as for *R_1_* and *R_2_* in several substructures, exhibiting higher rates of degeneration as compared to maturation. These differences in rates were statistically significant in several brainstem substructures for *R_1_*, *R_2_*, AxD, MD, and RD.

**Table 3:** Pearson correlation coefficient, rho, and FDR-corrected p-values reported across the 10 independent ROIs displayed in Figure 3A.

	***rho***	***p***
***MWF vs R_1_***	0.51	**p < 10^-6^**
***MWF vs R_2_***	0.25	**p < 10^-6^**
***MWF vs FA***	0.19	**p < 10^-6^**
***MWF vs MD***	-0.13	**p < 10^-5^**
***MWF vs RD***	-0.15	**p < 10^-6^**
***MWF vs AxD***	-0.07	**0.01**

## DISCUSSION

In this cross-sectional study, conducted on a large cohort of cognitively unimpaired subjects spanning an extended age range from 21 to 94 years, we combined several conventional and advanced quantitative MRI measurements, namely, longitudinal and transverse relaxation rates (*R_1_* and *R_2_*), DTI-derived indices, and MWF to characterize the maturation and degeneration phases of the human brainstem across the adult lifespan. This work was motivated by three additional emerging principles: first, the brainstem is involved in the early development of Alzheimer’s Disease and other neurodegenerative diseases. Second, the brainstem is an integrative brain center with key modulatory control over vital respiratory and cardiovascular physiology. Third, evidence suggests an association between age-related neurofunctional deficits, including decreased ability for spatial learning, memory impairment, perturbed auditory temporal processing speed, and diminished control of balance and gait with histological gross brainstem alterations [3, 25, 26]. While these postmortem and *ex-vivo* findings provide pivotal insights into our understanding of age-related alterations in the brainstem, it is difficult to perform these studies in conjunction with clinical neurophysiological assessments. Therefore, it is critical to establish specific and sensitive *in-vivo* imaging biomarkers that can help define the evolution of normal aging of the brainstem and characterize the heterogenous regional alterations that reflect the microstructural status of brainstem parenchyma. We believe that the present work provides a description of MR imaging biomarkers in the human brainstem that is unique in its incorporation of a large subject cohort of well-characterized cognitively normal adults and an exhaustive number of quantitative MR measures. These MR markers offer the possibility of probing tissue biophysical properties *in-vivo* and may help to further define the relationships between changes in cognition, function, and tissue microstructure.

Our investigation shows that the brainstem undergoes widespread changes throughout the human lifespan. In our previous study of MWF *vs.* age conducted on a cohort of 125 adult participants, we found a progressive increase in myelination from young adulthood through middle age followed by a progressive decrease through older age in a few brainstem substructures [15]. In fact, these quadratic associations were significant or close to statistical significance in only six of the fourteen brainstem regions evaluated, with only marginal trends in the other substructures. As noted, it was unclear whether the lack of quadratic trends reflected the statistical power of the study or rather mirrored a true monotonic time course of age-related decline in myelin content in the brainstem. In the present study, conducted on a larger cohort size (*N* = 140) and with an improved age distribution, our results showed that the quadratic trends of MWF with age were significant or close to significance in nine ROIs. This indicates that our previous study was underpowered. Perhaps more importantly, it reflects the fact that the myelination pattern of these brainstem substructures conforms to the inverted U-shaped relationship described in postmortem investigations and *in-vivo* MRI studies in the cerebrum [19, 27, 28]. These results indicate, however, that any quadratic trend in the brainstem is substantially less pronounced than the corresponding trends observed in the cerebrum. This may reflect functional and developmental differences between the brainstem and cerebrum. Nevertheless, we postulate that the observed myelination trends in the brainstem follow the proposed WM retrogenesis hypothesis (first-in-last-out). This suggests that early cerebral tissue development corresponds to a later age of degeneration. This principle has emerged from extensive studies that suggest that WM tracts myelinated later in maturation have thinner myelin sheets and are more susceptible to brain insults such as protein accumulation and iron deposition, while WM fascicles that develop earlier exhibit greater biophysiological stability [29–33]. In fact, the brainstem is one of the earliest structures to demonstrate significant myelination, with significant myelin content present even at the pre-term stage of development [34]. The autonomic and housekeeping functions of the brainstem are necessary from birth, while higher cognition can develop more slowly. Additionally, functional studies have indicated that phylogenetically conserved regions such as the brainstem are metabolically mature at birth while higher-functioning brain regions such as the cerebrum do not peak in energy demands and maturation until adulthood [35]. Our results further support this hypothesis since the significant quadratic associations with age are mostly confined to the superior anatomical regions of the brainstem (Table 1); these regions are more closely linked to higher-level function. This observation is further supported by postmortem findings that show an inferior-to-superior sequence of myelination in the brainstem [36]. However, more definitive definition of these patterns will require additional studies incorporating younger subjects. In addition, such studies would provide critical information regarding the estimated age of maximum myelination; these comments apply to studies of both the cerebrum and the brainstem.

It is also important to note that our choice of a linear regression model is consistent with visual inspection but may not reflect underlying age-related biological processes. Other models may serve equally well as descriptors of the data. Further work utilizing these models and, especially, longitudinal studies investigating myelination trajectories are required to establish time-course models that are accurate descriptors of underlying biological processes. Finally, we observed that the midbrain exhibited a rapid decline of MWF with age. This is consistent with morphometry-based studies that have shown that the midbrain, postulated to be the last brainstem region to be fully myelinated, is particularly susceptible to atrophy and iron deposition in comparison to other subdivisions such as the pons and medulla [10]. Iron deposition is known to be correlated with demyelination processes.

We found significant sex differences in myelin content in a limited number of brain regions before FDR correction, with women exhibiting higher myelin content than men. This finding is in accord with the consistent observation of higher cerebral myelin content in woman as compared to men [19, 27]. We also found that the MWF in women, as compared to men, shows more rapid reduction with age in several brain regions; this new finding indicates potential lines of investigation in larger cohorts. Indeed, the sex differences we observed in myelin are consistent with previous demonstrations that proliferation of oligodendrocytes and myelin proteins are regulated differently in males and females [37, 38]. A recent study suggests that sex steroids may influence this differential regulation, possibly modulating sex differences in repair [39].

Similar to MWF *vs*. age, our analysis suggests an inverted U-shaped association of *R_1_* and *R_2_* with age in most of the brainstem substructures examined. Although not specific to myelin content, *R_1_* and *R_2_* are very sensitive to myelin and have been extensively used to study brain developement and pathology [33, 40]. In fact, our Pearson correlation analysis indicated strong regional correlations between *R_1_* and MWF, and to a lesser extent, *R_2_* and MWF. This is expected due to the fact that *R_1_* is very sensitive to variation in lipid content [11, 33], the main constituent of myelin, while *R_2_* is further sensitive to other tissue properties including hydration, macromolecular content, temperature, iron content and flow, while being relatively less sensitive to lipids [11]. Thus, in addition to changes in myelin content, interpretation of conventional MRI metrics is greatly confounded by their sensitivity to other structural changes that may be taking place during brainstem maturation and degeneration, including changes in axonal density or axon caliber. As one example, morphometry-based studies have shown that certain brainstem substructures exhibit significant atrophy with aging, likely due to axonal loss [2, 10].

Although extensively documented in the cerebrum, to the best of our knowledge, no prior MRI-based study has sought to examine age- or sex-related differences in DTI indices in the human brainstem. The use of DTI indices in our study was driven by the fact that they are sensitive to the underlying microarchitectural status of the brain parenchyma and the degree and direction of water molecule mobility, yielding architectural information complementary to the other indices evaluated. Our novel results indicate a U-shaped relationship between most diffusivity metrics and age in most ROIs, with FA exhibiting an overall linear decline. These results are in accord with observed differences in DTI indices with age in the cerebral WM [41, 42], and further support the notion of brainstem maturation until middle age followed by more rapid degeneration. Moreover, consistent with other investigations, all DTI indices showed moderate-to-weak regional correlations with MWF [12, 27, 43–45]. This supports the notion that these indices, and especially FA and RD, may not serve as specific markers of myelin content. Figure 4 further supports this claim, clearly indicating that any single MRI parameter alone cannot describe the temporal and spatial maturation or degeneration process involved in senescence. This highlights the value of using multiple advanced and conventional quantitative MRI metrics that are specific and sensitive to distinct tissue features for clinical research and, potentially, for clinical application.

Our results are consistent with and complementary to the pioneering work of Lambert and colleagues, who reported a linear relationship between conventional quantitative MRI measures, including apparent transverse and longitudinal relaxation rates (R_2_^*^, *R_1_*) or magnetization transfer saturation and age in the brainstem [10]. These results were interpreted as indicators of axonal loss, demyelination, and increased iron accumulation. Our results, with a larger sample of more evenly distributed subjects, show that some of these trends may be better described by quadratic relationships with age. This also plausibly reflects the process of brainstem maturation followed by degeneration and is consistent with previous MR-based investigations and postmortem findings in the cerebrum [19, 27, 28, 33].

We observed well-defined spatial patterns for parameter values within the brainstem. We found progressively decreasing MWF, *R_1_*, *R_2_,* and increasing diffusivities from midbrain to medulla. We conjecture that these observed spatial variations reflect differences in tissue composition and function of the underlying substructures. The midbrain, the most superior anatomical division of the brainstem, contains tightly packed bundles of myelinated axons, such as the cerebral peduncle and the corticospinal tract, while the medulla, the inferior anatomical division, contains the cell bodies of cranial nerves and unmyelinated axons. This is consistent with anatomical studies and tractography investigations illustrating that certain myelinated tracts exhibit decreasing axonal or myelin densities as they extend from superior to inferior positions within the brainstem [46, 47].

Our results (Figures 3, 4) indicate that *R_1_* and MWF adhere to similar lifespan associations with age while DTI indices and *R_2_* demonstrate distinct lifespan trends in different brainstem regions. These nonuniform patterns of change observed in WM tracts and GM nuclei indicate that the biological underpinnings of brainstem tissue maturation and degeneration do not depend on the tissue composition only but are region dependent. This is consistent with multiple MRI studies that have shown that age-related tissue loss differs in extent and rate among brain regions [19, 27, 28, 33, 41, 48–52]. Moreover, we observed that the association with age of diffusivity indices and *R_2_* are distinct and, overall, peaked at a younger age decade as compared to MWF and *R_1_*. This likely reflects an earlier maturation of axonal bundles as compared to myelin sheets, followed by a rapid phase of axonal damage. Indeed, neurodevelopmental studies have shown that extensive brain alterations occur in the early stages of life associated with a reduction in plasticity due to axonal pruning [53, 54].

Interestingly, we observed that the variations in the diffusivity indices, that is, AxD, RD, and MD in various substructures during brainstem maturation (age range 20-40) sharply contrast with diffusivity changes in subsequent degeneration (age range 40-90). Specifically, the values of these indices in the midbrain, red nucleus, subthalamic nucleus, and substantia nigra more slowly approach a minimum in middle age while the degeneration phased afterward is significantly more rapid*.* Normal aging is a dynamic and complex process that attempts to approach a certain equilibrium of myelin and axonal generation or cellular turnover; however, we speculate that this dynamic process shifts towards a predominant degenerative phase at a certain age, perhaps in regions more prone to iron accumulation and lipid peroxidation such as the midbrain and its associated gray nuclei regions. Because the trends differ in MWF and *R_1_* with diffusivity indices and *R_2_*, this may also indicate that axons may degenerate at a faster rate than myelin. A more complete confirmation of this interpretation would require further histological or longitudinal studies.

Finally, we tested the gain-predicts-loss hypothesis in the specific context of brainstem maturation and degeneration. MRI-based studies have demonstrated support for this in the cerebrum [33], and have indicated that age-related processes are to a certain extent mirror-symmetric developmental processes in terms of time-course. In agreement with the observations in the cerebrum, our novel results provide support to the gain-predicts-loss hypothesis of tissue maturation and degeneration in the brainstem (Table 2). Indeed, MWF and *R_1_* both followed an inverted U-shape curve that was roughly symmetric with respect to age in most regions evaluated. Interestingly, this was not the case for the diffusivity indices and *R_2_*, for which the degenerative phase occurred significantly more rapidly than the maturation phase. We interpret these results as indicating that maturation and degeneration of myelin occurs in a more temporally symmetric fashion than axonal maturation and degeneration. Histological analyses, longitudinal studies, and studies involving younger participants are required to confirm this interpretation.

Although our work represents an extensive multi-modality MRI study using state-of-the-art methods on a large and well-controlled cohort of subjects, it nevertheless has important limitations. Our cohort, although relatively large and spanning a wide and well-sampled age range, does not include very young participants (< 20 years old); inclusion of younger participants may influence the quantification of the age trends [55]. However, this limitation derives from the exclusion criteria of the BLSA and GESTALT studies. We are currently developing recruitment strategies to extend the available age range. It is also important to note that while our study incorporated a larger cohort size (*N* = 140) with improved age distribution from our previous investigation [15], these age associations should still be interpreted with care as our results exhibit a large between subjects-heterogeneity. Further, optimal uniform sampling across all age intervals was not fully achieved; this may influence the overall interpretability of myelination or axonal density during senescence. Nevertheless, the improved age distribution in this present work with better age distribution allowed us to explore non-monatomic models with higher precision. In addition, as with all MRI sequences, there are numerous experimental and physiological limitations that may impact the derived parameter values. These include exchange between water pools, magnetization transfer between free water protons and macromolecules, iron content, and fiber crossing and fanning, which are not considered in all signal models of the evaluated MR measures. Moreover, Although we have conducted a careful examination of all ROIs, a certain degree of partial volume bias is unavoidable in the calculated parameter values. This issue highlights the challenge of accurately segmenting brainstem substructures; this is mainly due to their small size as well as the relatively poor contrast between regions. Indeed, due to the limited spatial resolution of the DW imaging datasets, contamination from CSF as well as partial volume issues may have been introduced. Indeed, the voxel volume of the DTI images is 2 times higher than that of the BMC-mcDESPOT images, leading to DTI metrics maps with much lower spatial resolution as compared to the MWF, *R_1_*, and *R_2_* maps. Furthermore, DW images are particularly susceptible to geometric distortions and partial volume effects from CSF, which may lead to unusually high diffusivity derived values. Although we mitigated, as much as possible, these limitations by eroding the ROIs, using manual interventions and image registration, further DTI studies with higher spatial resolution to reduce partial volume effects, and with images acquired with reversed phase-encode direction and higher number of *b_0_* volumes [56] to correct for susceptibility induced geometric distortions, are still needed. Finally, while we have provided evidence to demonstrate that MWF showed a moderate to weak regional correlation with DTI indices and a higher correlation with relaxation rates (Figure 3), part of the lowest correlation between MWF and DTI metrics could be explained by non-perfect image registration.

## MATERIALS AND METHODS

### Subjects

**Figure 6:**
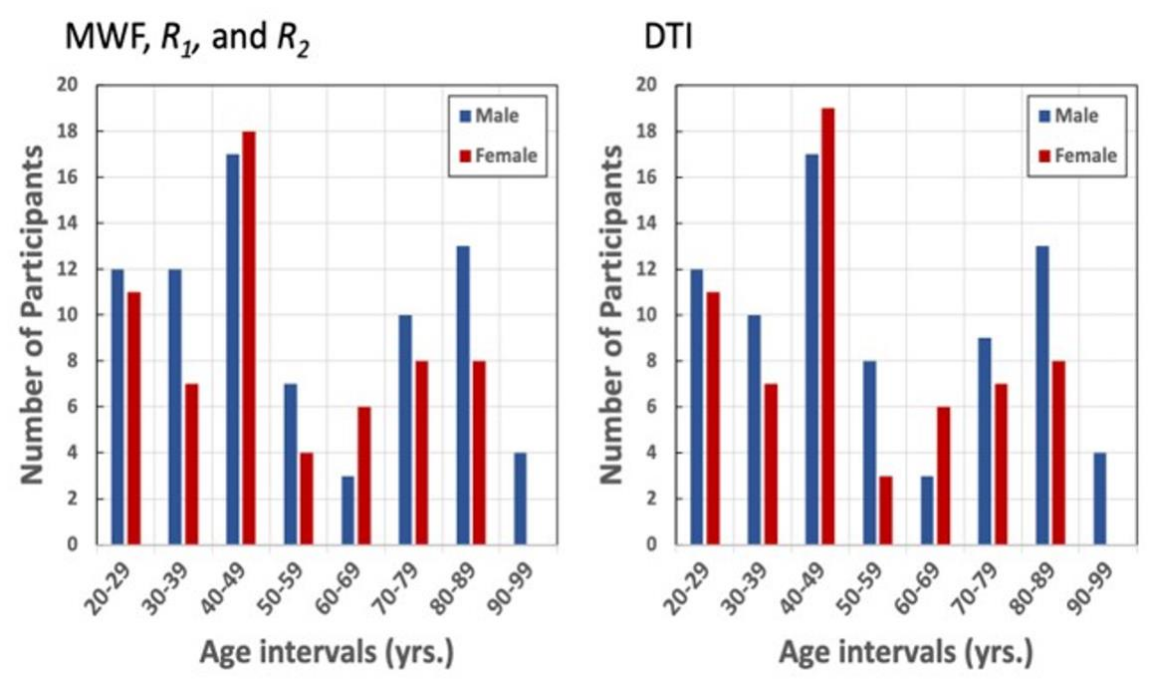
Number of participants per age decade and sex within the study cohorts for MWF, *R_1_*, *R_2_*, and DTI.

Cognitively unimpaired subjects were recruited from the Baltimore Longitudinal Study of Aging (BLSA), an ongoing study of normative aging in adults [57, 58], and the Genetic and Epigenetic Signatures of Translational Aging Laboratory Testing (GESTALT) study, an ongoing epidemiological, observational, and longitudinal study of adults. The BLSA is a longitudinal cohort study funded and conducted by the National Institute on Aging (NIA) Intramural Research Program (IRP). Established in 1958, the BLSA enrolls community-dwelling adults with no major chronic conditions or functional impairments. The GESTALT study is also a study of healthy volunteers, initiated in 2015, and funded and conducted by the NIA IRP. The goal of this study is to evaluate multiple biomarkers related to aging. Note that these studies do not differ in their population characteristics, so that combining subjects from them poses no difficulty. A detailed description of the cohort is presented in Table 4 and Figure 6. Age, MMSE, and years of education were not statistically significantly different between men and women.

**Table 4:** The characteristics of the participants’ cohort for each MR modality after removal of seven imaging datasets with technically limited scans caused by excessive motion.

	**Age (yrs.)**		**Sex**		**MMSE**	**Education (yrs.)**
	**Range**	**Mean ± SD**	**Men**	**Women**	**Mean ± SD**	**Mean ± SD**
MWF, *R_1_*, and *R_2_*	21 - 94	53.4 ± 21.4	78	62	28.5 ± 1.8	16.2 ± 2.8
DTI	21 - 94	53.2 ± 21.3	76	61	28.5 ± 1.8	16.1 ± 2.9

SD: standard deviation, MMSE: mini-mental state examination, MWF: myelin water fraction, R1 and R2: longitudinal and transverse relaxation rates, respectively, DTI: diffusion tensor imaging. For each modality, age, MMSE, and education years were not significantly different between men and women.

### MR imaging

All experiments were performed on a 3T whole body Philips MRI system (Achieva, Best, The Netherlands) using the internal quadrature body coil for transmission and an eight-channel phased-array head coil for reception. Experimental procedures were performed in compliance with our local Institutional Review Board, and all subjects provided written informed consent.

### Myelin water fraction, MWF, and longitudinal, R_1_, and transverse, R_2_, relaxation rates imaging and mapping

3D spoiled gradient recalled echo (SPGR) images were acquired with flip angles (FAs) of [2 4 6 8 10 12 14 16 18 20]°, echo time (TE) of 1.37 ms, and repetition time (TR) of ~5 ms, as well as 3D balanced steady state free precession (bSSFP) images acquired with FAs of [2 4 7 11 16 24 32 40 50 60]°, TE of 2.8 ms, and TR of 5.8 ms. The bSSFP images were acquired with radiofrequency excitation pulse phase increments of 0 or π in order to account for off-resonance effects [59]. All SPGR and bSSFP images were acquired with an acquisition matrix of 150 × 130 × 94, and voxel size of 1.6 mm × 1.6 mm × 1.6 mm. Further, we used the double-angle method (DAM) to correct for excitation radio frequency inhomogeneity [60]. For this, two fast spin-echo images were acquired with FAs of 45° and 90°, TE of 102 ms, TR of 3000 ms, and acquisition voxel size of 2.6 mm × 2.6 mm × 4 mm. All images were acquired with field-of-view (FOV) of 240 mm × 208 mm × 150 mm.

Using the FSL software [61], all SPGR, bSSFP, and DAM images were linearly registered to the averaged SPGR image over FAs and the derived transformation matrix was then applied to the original SPGR, bSSFP, and DAM images. Next, a whole-brain MWF map was generated for the remaining regions of interest using the BMC-mcDESPOT analysis from the registered SPGR, bSSFP, and DAM datasets [20, 62–64]. Briefly, BMC-mcDESPOT assumes a two-component system consisting of a slowly relaxing and a more rapidly relaxing component. The rapidly relaxing component corresponds to the signal of the water trapped within the myelin sheets while the slowly relaxing component corresponds to the intra and extra cellular waters. Analysis was performed explicitly accounting for nonzero TE as incorporated into the TE-corrected-mcDESPOT signal model [63]. A whole-brain *R_1_* map was generated from the registered SPGR and DAM datasets [65], and a whole-brain *R_2_* map was generated from the registered bSSFP and DAM datasets [65].

### Fractional anisotropy, FA, and mean, MD, radial, RD, and axial, AxD diffusivities imaging and mapping

The DTI protocol consisted of diffusion-weighted images (DWI) acquired with single-shot EPI, TR of 10,000 ms, TE of 70 ms, two *b*-values of 0 and 700 s/mm^2^, with the latter encoded in 32 directions, acquisition matrix of 120 × 104 × 75, and acquisition voxel size of 2 mm × 2 mm × 2 mm. Two images at *b* = 0 s/mm^2^ were acquired. All images were acquired with FOV of 240 mm × 208 mm × 150 mm. The DW images were corrected for eddy current and motion effects using affine registration tools as implemented in FSL. Then, the DW images were registered to the averaged DW images obtained with *b* = 0 s/mm^2^, and the derived transformation matrix was then applied to the original DW images. Calculation of eigenvalues from the DTI dataset was conducted on the registered DW images using the *DTIfit* tool implemented in FSL, which independently fits diffusion tensors to each voxel and provides voxel-wise eigenvalue maps. Finally, these derived eigenvalue maps were used to calculate FA, RD, MD and AxD, as conventional.

### Image registration

The scalp, ventricles, and other nonparenchymal regions within the images were eliminated using FSL. The SPGR image averaged over FAs for each participant was registered using nonlinear registration to the Montreal Neurological Institute (MNI) standard space image and the derived transformation matrix was then applied to the MWF, *R_1_*, and *R_2_* maps for that corresponding participant. Similarly, for each participant, the averaged DW image obtained at *b* = 0 s/mm^2^ was nonlinearly registered to the MNI atlas and the calculated matrix of transformation matrix was then applied to the corresponding FA, RD, MD, and AxD maps.

### ROIs segmentation

Fourteen brainstem structures were chosen as regions of interest (ROIs) from the Johns Hopkins University (JHU) ICGM-DTI 81 atlas [66, 67] and the Talairach structural atlas provided in FSL to cover all the ROIs for this investigation (Figure 5). ROIs were manually adjusted on single subject-level when needed to reduce partial volume effects and imperfect image registration. Six independent, non-overlapping, white matter ROIs were derived from the JHU atlas; these were the superior cerebellar peduncle, middle cerebellar peduncle, inferior cerebellar peduncle, corticospinal tract, lemniscus tract, and pontine tract. Four additional white matter ROIs were derived from the Talairach atlas corresponding to the midbrain, pons, medulla, and whole brainstem white matter, while three independent, non-overlapping, gray matter ROIs were derived from the same atlas corresponding to the substantia nigra, red nucleus, and subthalamic nucleus. Of note, the pons, medulla, midbrain, and whole brainstem WM ROIs obtained from the Talairach structural atlas encompass all WM within the 10 anatomical subdivisions. The GM within these ROIs was excluded.

Most ROIs were eroded to reduce partial volume effects and imperfect image registration using a kernel box of 2 voxels × 2 voxels × 2 voxels with the FSL tool *fslmaths*. The JHU-ICBM atlas ROIs were further superimposed onto the Harvard-Oxford brainstem atlas in FSL to confine the ROIs to the brainstem while avoiding anatomic overlap with nearby brain regions. For each ROI, the mean MWF, *R_1_*, *R_2_*, FA, MD, RD, and AxD values were calculated for each subject from the normalized space, as well as the mean and standard deviation (SD) parameter values averaged over participants for each age-interval described by Figure 6. We note that the parameter maps calculation, image registration, and ROI segmentation were performed blinded to the participants’ age, sex, and cognitive status.

### Statistical analysis

### Effect of age and sex

For each ROI, a multi linear regression model was evaluated using the mean ROI values for MWF, *R_1_*, *R_2_*, FA, MD, RD, or AxD as the dependent variable and sex and age as the independent variables. Examination of Figure 2 indicates that MWF, relaxation rates, and DTI outcomes (RD, MD, and AxD) follow quadratic associations with age in several ROIs. Therefore, we incorporated a quadratic age term, age^2^, in the regression model. The initial model incorporated interactions between sex and age and sex and age^2,^ which were removed if not significant, with the resulting parsimonious model then evaluated without these nonsignificant interaction terms.

### Parameters correlation with MWF

It has been widely assumed that the relaxivity rates and DTI outcomes (especially FA and RD) could serve as specific metrics to probe changes in myelin content with neurodevelopment or pathology [40, 68, 69]. Here, for each ROI, we tested this assumption using Pearson correlation by correlating each derived parameter, that is, *R_1_*, *R_2_*, FA, MD, RD, or AxD to MWF; this later represents a more specific proxy of myelin content [70–72].

### Maturation and degeneration phases

To highlight microstructural maturation and degeneration in the human brainstem across the adult life span, we plotted the Z-scores for MWF, relaxation rates, and DTI indices values as a function of age in six substructures, including three WM and three GM, selected based on their demonstration of significant quadratic associations between age and MWF, relaxivity rates, and DTI indices. FA was excluded from our analysis since this metric did not demonstrate significant nonlinear associations with age in most regions examined. We note that the diffusivity indices were inverted for easier comparison.

### Evaluating the gain-predicts-loss hypothesis

The gain-predicts-loss hypothesis suggests that the rate of tissue gain during maturation at younger age will equal the rate of tissue loss during degeneration at older age [33]. To test this paradigm, we characterized the lifespan changes in MWF, relaxation rates, or DTI indices for each ROI by fitting the standardized data to a piecewise linear model [33]. This model consisted of two segments, with the first corresponding to the maturation phase and the second to the degeneration phase. The point of transition was defined as the maximum or minimum, as appropriate, of the parameter under consideration. The slopes of these two segments defined the rates of maturation and degeneration; their absolute values were compared statistically for each ROI investigated.

In all cases, the threshold for statistical significance was taken as *p* < 0.05 after correction for multiple ROI comparisons using the false discovery rate (FDR) method [73, 74].

## Supplementary Material

Supplementary Figures

Supplementary Tables
