# Supplementary material for: Maturation and degeneration of the human brainstem across the adult lifespan
Source: Aging (Albany NY). 2021 Jun 11;13(11):14862–91. doi: 10.18632/aging.203183 (PMC8221341; doi:10.18632/aging.203183)
Supplement: Supplementary Figures [file aging-13-203183-s001.pdf]

SUPPLEMENTARY FIGURES

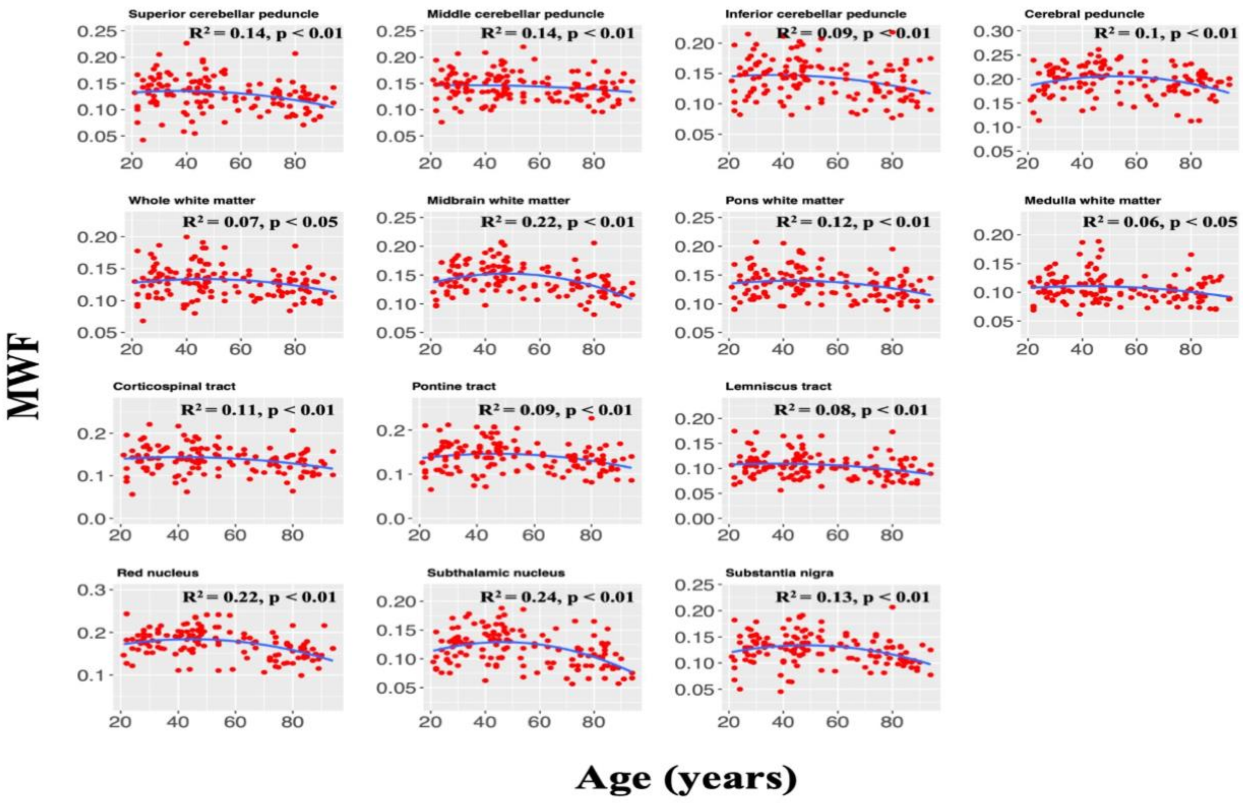

Supplementary Figure 1. Plots illustating regional MWF values as a function of age white matter and deep grey nuclei substructures in the brainstem with a sample size of *N* = 140. For each ROI, the coefficient of determination, R^2, and the significance of the linear regression model, *p*, are reported. Most regions investigated show an inverted U-shaped trend in MWF with age while exhibiting variation in these trends.

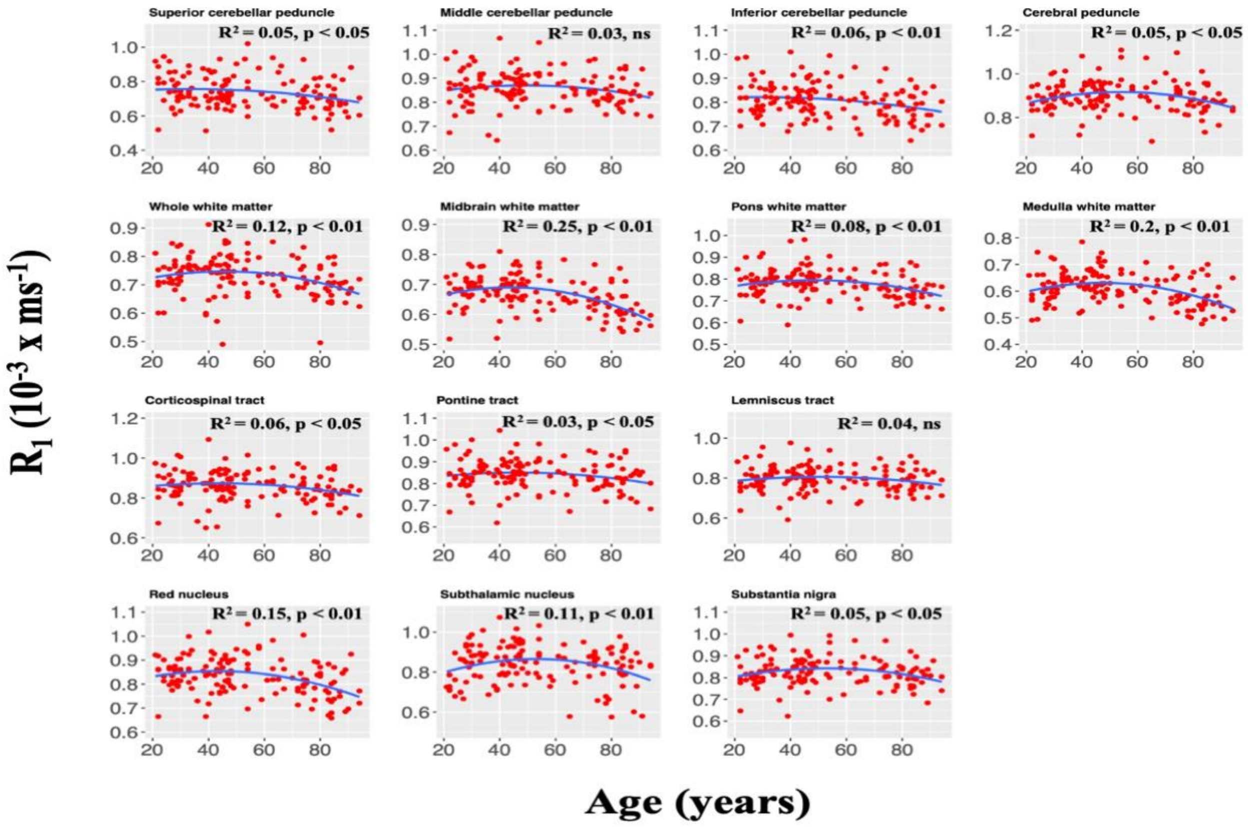

Supplementary Figure 2. Plots illustrating regional R_1 values as a function of age for white matter and deep grey nuclei substructures in the brainstem with a sample size of *N* = 140. For each ROI, the coefficient of determination, R^2, and the significance of the linear regression model, *p*, are reported. All regions investigated show an inverted U-shaped trend in R_1 with age while exhibiting variation in these trends.

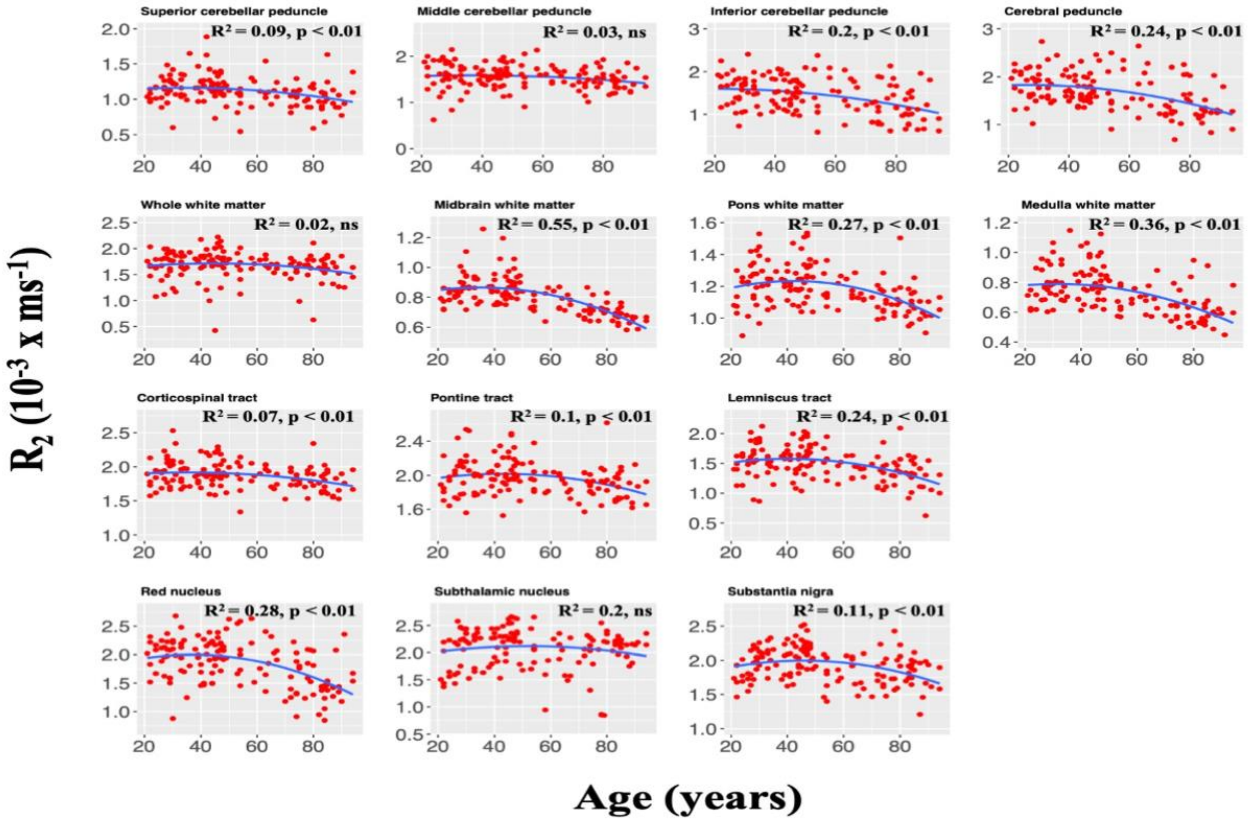

Supplementary Figure 3. Plots illustrating regional R_2 values as a function of age for white matter and deep grey nuclei substructures in the brainstem with a sample size of N = 140. For each ROI, the coefficient of determination, R^2, and the significance of the linear regression model, p, are reported. All regions investigated show an inverted U-shaped trend in R_2 with age while exhibiting variation in these trends.

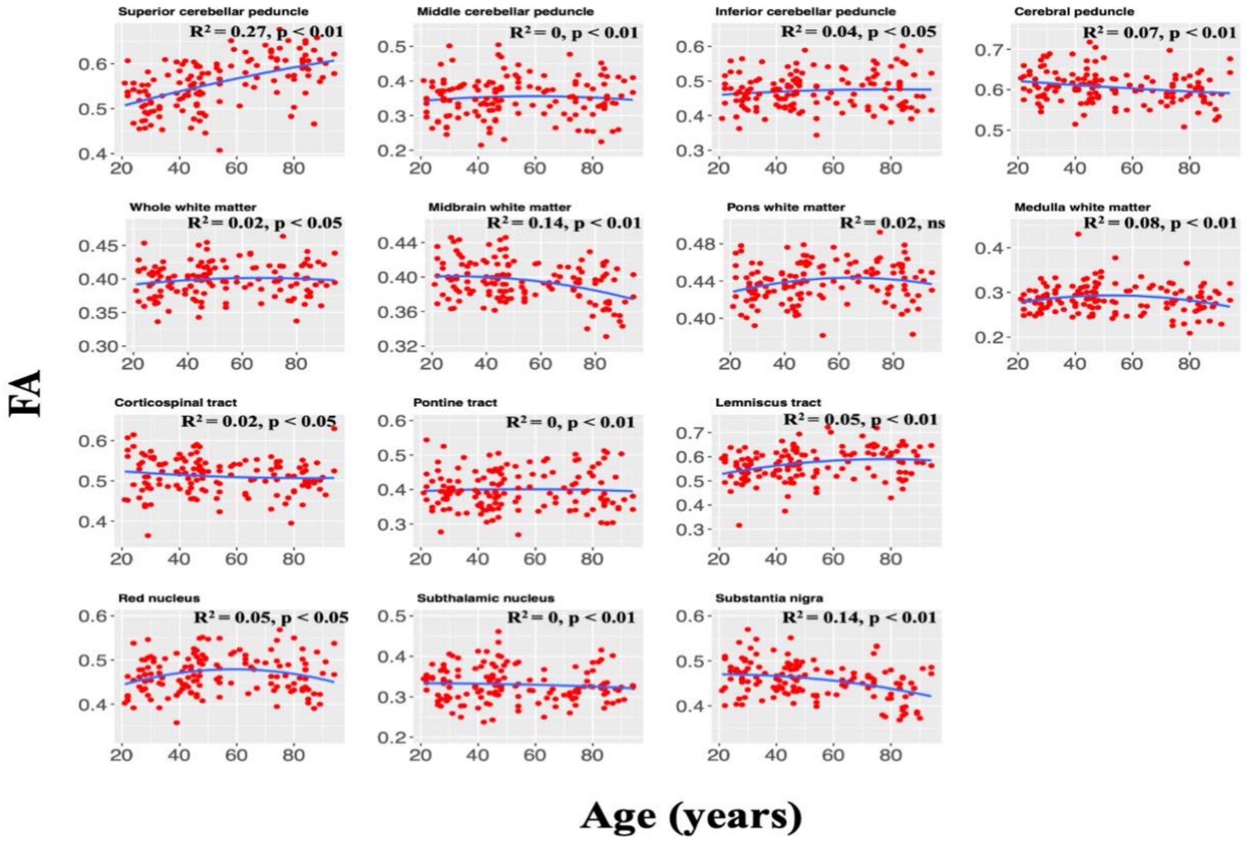

Supplementary Figure 4. Plots illustrating regional FA values as a function of age for white matter and deep grey nuclei substructures in the brainstem with a sample size of *N* = 137. For each ROI, the coefficient of determination, R^2, and the significance of the linear regression model, *p*, are reported. The red nucleus, whole WM midbrain WM, pons WM, medulla WM, substantia nigra, and lemniscus tract exhibited a quadratic trend of age with FA. The cerebral peduncle and superior cerebellar peduncle showed a linear trend of FA with age while the remaining regions did not show a significant association between FA and age.

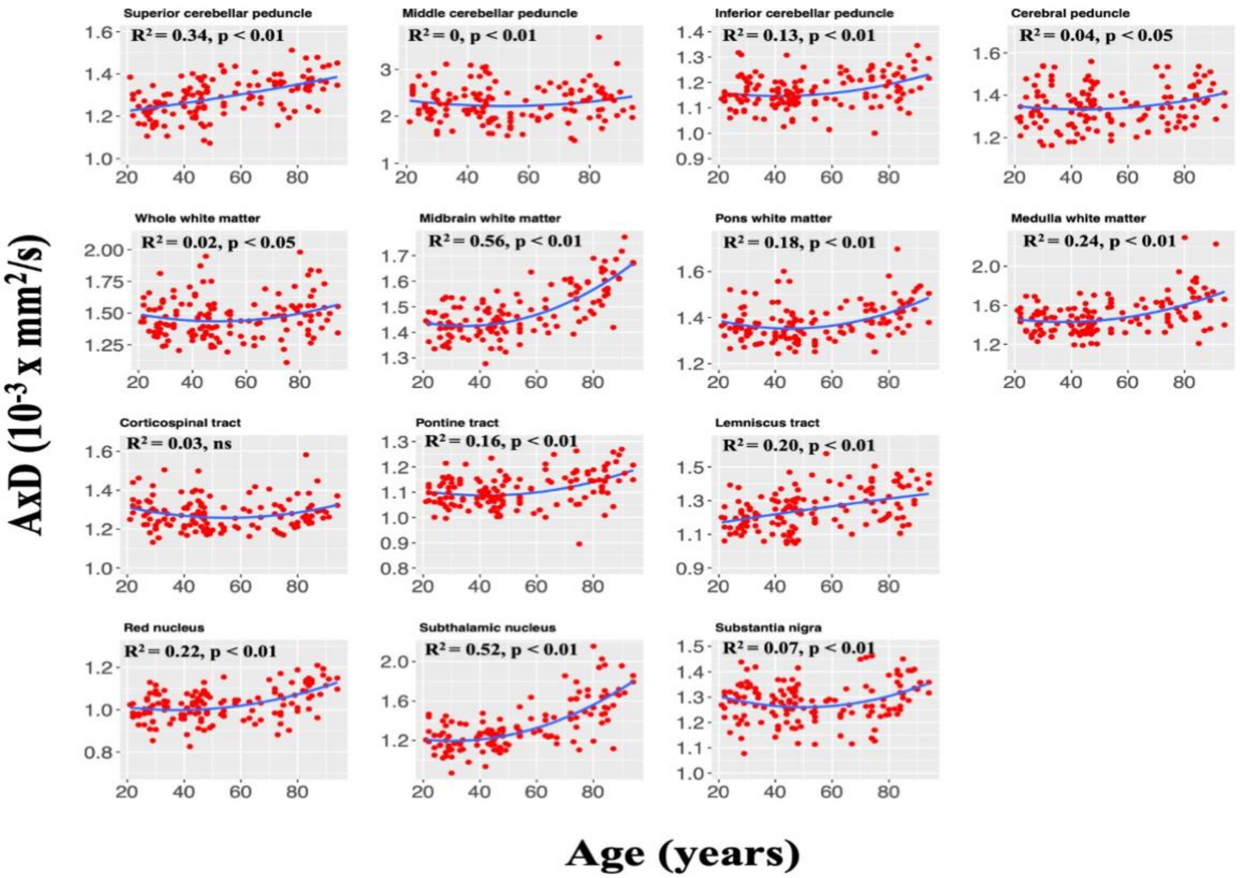

Supplementary Figure 5. Plots illustrating regional AD values as a function of age for white matter and deep grey nuclei substructures in the brainstem with a sample size of *N* = 137. For each ROI, the coefficient of determination, R^2, and the significance of the linear regression model, *p*, are reported. A majority of the regions investigated show a U-shaped trend of AxD with age while exhibiting variation in these trends.

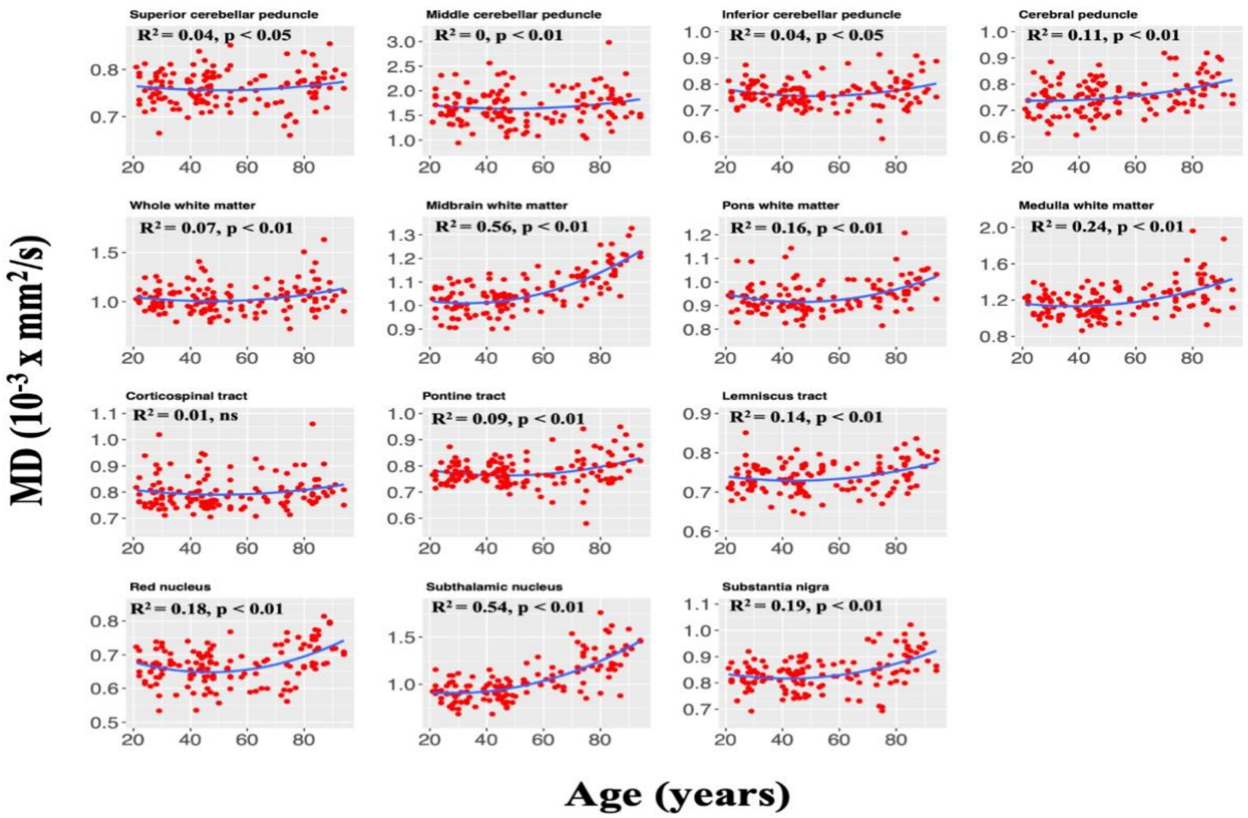

Supplementary Figure 6. Plots illustrating regional MD values as a function of age for white matter and deep grey nuclei substructures in the brainstem with a sample size of *N* = 137. For each ROI, the coefficient of determination, R^2, and the significance of the linear regression model, *p*, are reported. A majority of the regions investigated show a U-shaped trend of MD with age while exhibiting variation in trends.

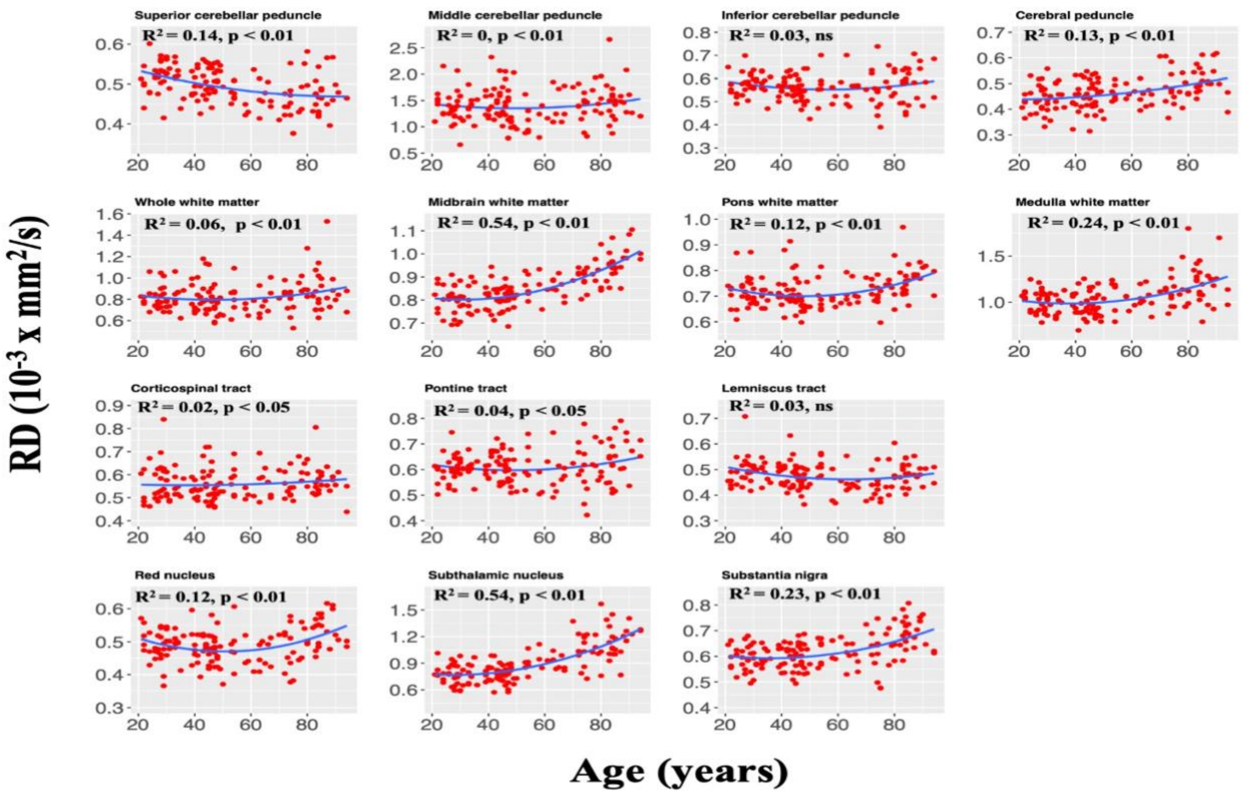

Supplementary Figure 7. Plots illustrating regional RD values as a function of age for white matter and deep grey nuclei substructures in the brainstem with a sample size of *N* = 137. For each ROI, the coefficient of determination, R^2, and the significance of the linear regression model, *p*, are reported. A majority of the regions investigated show a U-shaped trend of RD with age while exhibiting variation in trends.
