# Supplementary material for: Maturation and degeneration of the human brainstem across the adult lifespan
Source: Aging (Albany NY). 2021 Jun 11;13(11):14862–91. doi: 10.18632/aging.203183 (PMC8221341; doi:10.18632/aging.203183)
Supplement: Supplementary Tables [file aging-13-203183-s002.pdf]

SUPPLEMENTARY TABLES

Supplementary Table 1. Regional mean ± standard deviation (SD) of derived MWF values averaged over participants within each age decade.

	Mean ± SD MWF values								
	20-29 yrs.	30-39 yrs.	40-49 yrs.	50-59 yrs.	60-69 yrs.	70-79 yrs.	80-89 yrs.	90-99 yrs.	20-99 yrs.
Superior cerebellar peduncle	0.13±0.03	0.14±0.03	0.14±0.03	0.14±0.03	0.12±0.01	0.11±0.02	0.12±0.03	0.12±0.01	0.13±0.03
Middle cerebellar peduncle	0.15±0.03	0.14±0.03	0.15±0.03	0.16±0.03	0.13±0.02	0.14±0.02	0.14±0.02	0.15±0.02	0.14±0.03
Inferior cerebellar peduncle	0.14±0.04	0.15±0.03	0.15±0.03	0.15±0.03	0.12±0.03	0.13±0.02	0.13±0.03	0.15±0.03	0.14±0.03
Cerebral peduncle	0.19±0.03	0.20±0.02	0.21±0.03	0.20±0.04	0.19±0.03	0.19±0.03	0.19±0.03	0.19±0.01	0.20±0.03
Corticospinal tract	0.14±0.04	0.14±0.03	0.15±0.03	0.15±0.03	0.13±0.02	0.13±0.02	0.13±0.03	0.13±0.02	0.14±0.03
Pontine tract	0.14±0.03	0.14±0.03	0.15±0.03	0.14±0.03	0.13±0.02	0.13±0.02	0.13±0.03	0.13±0.03	0.14±0.03
Lemniscus tract	0.11±0.03	0.11±0.02	0.11±0.03	0.11±0.03	0.10±0.02	0.10±0.02	0.10±0.03	0.10±0.01	0.11±0.03
Whole white matter	0.13±0.03	0.13±0.02	0.14±0.03	0.14±0.02	0.12±0.02	0.12±0.02	0.12±0.02	0.12±0.02	0.13±0.02
Midbrain white matter	0.14±0.02	0.15±0.02	0.16±0.03	0.14±0.02	0.14±0.02	0.13±0.02	0.13±0.03	0.13±0.01	0.14±0.03
Pons white matter	0.14±0.04	0.14±0.02	0.15±0.03	0.14±0.02	0.12±0.02	0.13±0.02	0.13±0.02	0.13±0.02	0.14±0.02
Medulla white matter	0.11±0.02	0.11±0.02	0.12±0.03	0.10±0.01	0.10±0.02	0.10±0.02	0.10±0.02	0.11±0.02	0.11±0.02
Red nucleus	0.17±0.03	0.18±0.01	0.19±0.03	0.19±0.04	0.17±0.02	0.15±0.03	0.15±0.02	0.17±0.03	0.17±0.03
Subthalamic nucleus	0.11±0.03	0.13±0.02	0.13±0.03	0.12±0.03	0.12±0.02	0.10±0.03	0.11±0.03	0.08±0.01	0.12±0.03
Substantia nigra	0.12±0.03	0.13±0.03	0.14±0.03	0.13±0.03	0.13±0.02	0.12±0.02	0.11±0.03	0.11±0.03	0.13±0.03

The last column shows the results derived across the whole age range.

Supplementary Table 2. Regional mean ± standard deviation (SD) of derived R_1 values averaged over participants within each age decade.

	Mean ± SD R_1 x 10^{-3} values (ms^{-1})								
	20-29 yrs.	30-39 yrs.	40-49 yrs.	50-59 yrs.	60-69 yrs.	70-79 yrs.	80-89 yrs.	90-99 yrs.	20-99 yrs.
Superior cerebellar peduncle	0.77±0.11	0.74±0.09	0.74±0.07	0.80±0.13	0.77±0.10	0.72±0.08	0.69±0.08	0.70±0.12	0.74±0.09
Middle cerebellar peduncle	0.85±0.08	0.85±0.09	0.87±0.06	0.89±0.08	0.85±0.06	0.85±0.06	0.84±0.06	0.84±0.07	0.86±0.07
Inferior cerebellar peduncle	0.82±0.07	0.81±0.06	0.82±0.07	0.83±0.09	0.79±0.08	0.78±0.06	0.78±0.07	0.78±0.06	0.81±0.07
Cerebral peduncle	0.88±0.06	0.90±0.06	0.91±0.06	0.94±0.09	0.89±0.10	0.91±0.07	0.87±0.06	0.86±0.02	0.90±0.07
Corticospinal tract	0.88±0.07	0.84±0.08	0.87±0.08	0.89±0.07	0.85±0.06	0.85±0.06	0.84±0.06	0.81±0.06	0.86±0.07
Pontine tract	0.84±0.07	0.85±0.08	0.85±0.07	0.86±0.08	0.82±0.07	0.83±0.06	0.83±0.07	0.79±0.07	0.84±0.07
Lemniscus tract	0.79±0.06	0.80±0.08	0.81±0.06	0.82±0.06	0.78±0.06	0.78±0.05	0.79±0.06	0.77±0.04	0.80±0.06
Whole white matter	0.73±0.06	0.74±0.06	0.75±0.08	0.75±0.06	0.73±0.05	0.72±0.05	0.70±0.07	0.68±0.04	0.73±0.07
Midbrain white matter	0.67±0.06	0.69±0.05	0.69±0.05	0.68±0.06	0.67±0.04	0.64±0.05	0.62±0.06	0.56±0.02	0.67±0.06
Pons white matter	0.77±0.06	0.78±0.07	0.80±0.07	0.79±0.06	0.76±0.05	0.76±0.05	0.76±0.06	0.73±0.09	0.78±0.06
Medulla white matter	0.60±0.06	0.63±0.05	0.64±0.05	0.62±0.04	0.61±0.06	0.59±0.06	0.58±0.06	0.55±0.06	0.61±0.06
Red nucleus	0.84±0.06	0.85±0.07	0.85±0.06	0.89±0.10	0.85±0.06	0.81±0.07	0.77±0.07	0.78±0.09	0.83±0.08
Subthalamic nucleus	0.80±0.09	0.85±0.09	0.88±0.08	0.84±0.09	0.82±0.10	0.82±0.09	0.11±0.03	0.77±0.11	0.84±0.09
Substantia nigra	0.81±0.06	0.83±0.07	0.84±0.05	0.85±0.09	0.84±0.06	0.82±0.06	0.81±0.05	0.81±0.05	0.83±0.06

The last column shows the results derived across the whole age range.

Supplementary Table 3. Regional mean ± standard deviation (SD) of derived R_2 values averaged over participants within each age decade.

	Mean ± SD R_2 x 10^{-3} values (ms^{-1})								
	20-29 yrs.	30-39 yrs.	40-49 yrs.	50-59 yrs.	60-69 yrs.	70-79 yrs.	80-89 yrs.	90-99 yrs.	20-99 yrs.
Superior cerebellar peduncle	1.25±0.47	1.16±0.47	1.02±0.33	1.24±0.56	1.16±0.42	0.99±0.42	0.79±0.25	0.98±0.46	1.06±0.43
Middle cerebellar peduncle	1.59±0.33	1.57±0.30	1.58±0.20	1.64±0.33	1.60±0.13	1.48±0.22	1.46±0.21	1.51±0.16	1.55±0.26
Inferior cerebellar peduncle	1.56±0.37	1.63±0.36	1.51±0.30	1.57±0.54	1.46±0.38	1.27±0.45	1.12±0.35	1.135±0.44	1.43±0.42
Cerebral peduncle	1.78±0.32	1.92±0.34	1.74±0.26	1.78±0.50	1.81±0.44	1.45±0.42	1.33±0.29	1.31±0.32	1.67±0.40
Corticospinal tract	1.89±0.18	1.93±0.21	1.93±0.18	1.87±0.22	1.88±0.13	1.80±0.13	1.78±0.19	0.82±0.11	1.87±0.19
Pontine tract	1.97±0.20	2.02±0.24	2.05±0.20	1.99±0.17	1.92±0.10	1.88±0.14	1.88±0.22	1.86±0.17	1.97±0.21
Lemniscus tract	1.53±0.27	1.57±0.27	1.59±0.26	1.54±0.23	1.42±0.14	1.35±0.20	1.33±0.30	1.23±0.22	1.49±0.28
Whole white matter	1.68±0.25	1.69±0.23	0.75±0.08	1.67±0.23	0.73±0.05	1.61±0.19	1.60±0.30	1.55±0.22	1.67±0.27
Midbrain white matter	0.84±0.07	0.90±0.12	0.88±0.10	0.77±0.07	0.78±0.05	0.71±0.06	0.69±0.06	0.64±0.03	0.80±0.12
Pons white matter	1.20±0.13	1.23±0.14	1.25±0.13	1.20±0.10	1.17±0.06	1.10±0.08	1.10±0.13	1.08±0.04	1.18±0.13
Medulla white matter	0.76±0.11	0.82±0.14	0.82±0.13	0.69±0.08	0.69±0.09	0.63±0.09	0.61±0.12	0.60±0.12	0.73±0.14
Red nucleus	1.96±0.27	2.00±0.18	1.97±0.27	2.10±0.44	1.93±0.31	1.61±0.36	1.42±0.30	1.66±0.43	1.85±0.40
Subthalamic nucleus	1.97±0.38	2.11±0.31	1.74±0.34	1.90±0.43	1.70±0.10	1.85±0.51	2.13±0.16	2.21±0.09	2.07±0.37
Substantia nigra	1.87±0.21	2.05±0.18	2.09±0.22	1.77±0.21	1.84±0.23	1.80±0.25	1.84±0.23	1.76±0.16	1.92±0.25

The last column shows the results derived across the whole age range.

Supplementary Table 4. Regional mean ± standard deviation (SD) of derived FA values averaged over participants within each age decade.

	Mean ± SD FA values								
	20-29 yrs.	30-39 yrs.	40-49 yrs.	50-59 yrs.	60-69 yrs.	70-79 yrs.	80-89 yrs.	90-99 yrs.	20-99 yrs.
Superior cerebellar peduncle	0.52±0.04	0.52±0.04	0.54±0.04	0.57±0.06	0.58±0.04	0.60±0.05	0.59±0.05	0.59±0.02	0.56±0.05
Middle cerebellar peduncle	0.34±0.05	0.37±0.05	0.35±0.06	0.39±0.04	0.35±0.04	0.35±0.06	0.34±0.06	0.38±0.07	0.35±0.06
Inferior cerebellar peduncle	0.47±0.04	0.47±0.04	0.47±0.04	0.47±0.07	0.47±0.05	0.48±0.05	0.47±0.06	0.50±0.07	0.47±0.05
Cerebral peduncle	0.61±0.04	0.63±0.03	0.61±0.04	0.20±0.04	0.60±0.03	0.60±0.04	0.59±0.03	0.61±0.06	0.20±0.03
Corticospinal tract	0.51±0.06	0.52±0.04	0.52±0.04	0.50±0.03	0.51±0.04	0.50±0.05	0.50±0.03	0.53±0.06	0.51±0.04
Pontine tract	0.40±0.06	0.40±0.04	0.39±0.05	0.41±0.06	0.41±0.05	0.41±0.05	0.39±0.07	0.40±0.06	0.14±0.03
Lemniscus tract	0.54±0.07	0.55±0.04	0.57±0.06	0.57±0.08	0.58±0.05	0.62±0.06	0.57±0.06	0.61±0.04	0.57±0.07
Whole white matter	0.39±0.03	0.40±0.03	0.14±0.03	0.40±0.02	0.40±0.02	0.40±0.03	0.40±0.03	0.41±0.02	0.40±0.03
Midbrain white matter	0.40±0.02	0.40±0.02	0.40±0.02	0.39±0.02	0.40±0.01	0.39±0.02	0.38±0.02	0.37±0.02	0.39±0.02
Pons white matter	0.43±0.02	0.44±0.01	0.44±0.02	0.44±0.03	0.45±0.01	0.45±0.02	0.44±0.02	0.44±0.01	0.44±0.02
Medulla white matter	0.23±0.02	0.29±0.02	0.30±0.04	0.29±0.04	0.29±0.03	0.29±0.04	0.27±0.03	0.28±0.03	0.29±0.03
Red nucleus	0.50±0.04	0.47±0.04	0.19±0.03	0.48±0.04	0.48±0.03	0.47±0.04	0.45±0.04	0.49±0.03	0.47±0.04
Subthalamic nucleus	0.33±0.03	0.34±0.05	0.13±0.03	0.33±0.04	0.30±0.03	0.31±0.03	0.33±0.04	0.32±0.01	0.33±0.04
Substantia nigra	0.46±0.04	0.46±0.03	0.14±0.03	0.45±0.03	0.46±0.02	0.46±0.04	0.42±0.03	0.45±0.05	0.46±0.04

The last column shows the results derived across the whole age range.

Supplementary Table 5. Regional mean ± standard deviation (SD) of derived AxD values averaged over participants within each age decade.

	Mean ± SD AxD x 10^{-3} values (mm^2/s)								
	20-29 yrs.	30-39 yrs.	40-49 yrs.	50-59 yrs.	60-69 yrs.	70-79 yrs.	80-89 yrs.	90-99 yrs.	20-99 yrs.
Superior cerebellar peduncle	1.24±0.07	1.24±0.07	1.27±0.08	1.34±0.08	1.31±0.05	1.33±0.09	1.36±0.06	1.40±0.05	1.29±0.09
Middle cerebellar peduncle	2.29±0.29	2.23±0.37	2.32±0.40	1.94±0.27	2.21±0.03	2.27±0.37	2.42±0.43	2.20±0.20	2.27±0.38
Inferior cerebellar peduncle	1.15±0.07	1.16±0.05	1.14±0.05	1.14±0.07	1.19±0.06	1.18±0.07	1.19±0.06	1.26±0.07	1.12±0.07
Cerebral peduncle	1.33±0.08	1.35±0.13	1.35±0.09	1.30±0.07	1.30±0.05	1.36±0.09	1.40±0.08	1.38±0.06	1.35±0.09
Corticospinal tract	1.30±0.08	1.26±0.08	1.27±0.18	1.23±0.04	1.29±0.08	1.27±0.07	1.30±0.09	1.31±0.04	1.28±0.08
Pontine tract	1.09±0.05	1.10±0.05	1.09±0.05	1.09±0.04	1.12±0.08	1.17±0.08	1.15±0.06	1.20±0.05	1.11±0.07
Lemniscus tract	1.18±0.08	1.20±0.07	1.22±0.11	1.31±0.12	1.26±0.10	1.31±0.10	1.29±0.12	1.41±0.03	1.25±0.11
Whole white matter	1.47±0.13	1.45±0.14	1.45±0.17	1.38±0.10	1.43±0.13	1.46±0.15	1.56±0.19	1.46±0.08	1.47±0.16
Midbrain white matter	1.42±0.06	1.44±0.05	1.43±0.06	1.47±0.08	1.47±0.06	1.58±0.06	1.59±0.07	1.71±0.04	1.48±0.09
Pons white matter	1.38±0.06	1.35±0.06	1.36±0.08	1.34±0.06	1.37±0.05	1.40±0.06	1.44±0.08	1.49±0.06	1.38±0.08
Medulla white matter	1.45±0.13	1.44±0.09	1.40±0.13	1.51±0.13	1.49±0.10	1.57±0.15	1.64±0.23	1.76±0.30	1.50±0.18
Red nucleus	0.99±0.06	1.02±0.06	1.01±0.07	1.02±0.06	1.00±0.05	1.03±0.08	1.10±0.07	1.13±0.02	1.03±0.08
Subthalamic nucleus	1.22±0.13	1.16±0.13	1.22±0.11	1.34±0.10	1.30±0.10	1.53±0.20	1.61±0.25	1.82±0.10	1.34±0.24
Substantia nigra	1.27±0.08	1.31±0.06	1.27±0.07	1.24±0.05	1.23±0.06	1.28±0.10	1.33±0.07	1.34±0.02	1.28±0.08

The last column shows the results derived across the whole age range.

Supplementary Table 6. Regional mean ± standard deviation (SD) of derived MD values averaged over participants within each age decade.

	Mean ± SD MD x 10^{-3} values (mm^2/s)								
	20-29 yrs.	30-39 yrs.	40-49 yrs.	50-59 yrs.	60-69 yrs.	70-79 yrs.	80-89 yrs.	90-99 yrs.	20-99 yrs.
Superior cerebellar peduncle	0.77±0.03	0.76±0.03	076±0.03	0.76±0.04	0.75±0.03	0.74±0.05	0.77±0.04	0.79±0.01	0.76±0.04
Middle cerebellar peduncle	1.69±0.27	1.61±0.03	1.70±0.04	1.41±0.26	1.65±0.30	1.72±0.32	1.82±0.39	1.61±0.18	1.68±0.34
Inferior cerebellar peduncle	0.77±0.04	0.77±0.04	0.76±0.04	0.75±0.04	0.77±0.05	0.76±0.07	0.79±0.06	0.80±0.05	0.77±0.05
Cerebral peduncle	0.73±0.05	0.74±0.07	0.75±0.06	0.74±0.03	0.73±0.04	0.78±0.07	0.81±0.05	0.78±0.07	0.76±0.06
Corticospinal tract	0.81±0.07	0.78±0.05	0.79±0.06	0.78±0.05	0.80±0.06	0.80±0.05	0.82±0.07	0.80±0.03	0.80±0.06
Pontine tract	0.77±0.04	0.77±0.02	0.77±0.04	0.75±0.05	0.77±0.06	0.78±0.08	0.81±0.06	0.83±0.04	0.78±0.05
Lemniscus tract	0.73±0.04	0.74±0.03	0.73±0.04	0.75±0.03	0.73±0.03	0.73±0.03	0.76±0.04	0.79±0.01	0.74±0.04
Whole white matter	1.04±0.10	1.01±0.11	1.01±0.14	0.97±0.11	1.00±0.12	1.04±0.13	1.12±0.20	1.02±0.08	1.03±0.14
Midbrain white matter	1.01±0.06	1.02±0.06	1.01±0.05	1.06±0.06	1.06±0.04	1.11±0.04	1.16±0.06	1.26±0.05	1.06±0.08
Pons white matter	0.94±0.06	0.91±0.04	0.92±0.07	0.91±0.05	0.93±0.05	0.95±0.05	0.99±0.07	1.02±0.05	0.94±0.07
Medulla white matter	1.16±0.12	1.14±0.09	1.11±0.13	1.21±0.14	1.19±0.11	1.27±0.15	1.34±0.22	1.43±0.28	1.20±0.18
Red nucleus	0.65±0.05	0.68±0.04	0.66±0.05	0.65±0.05	0.64±0.04	0.67±0.07	0.72±0.05	0.71±0.01	0.67±0.06
Subthalamic nucleus	0.93±0.11	0.89±0.11	0.92±0.09	1.04±0.12	1.04±0.08	1.23±0.19	1.29±0.21	1.47±0.07	1.04±0.22
Substantia nigra	0.82±0.05	0.83±0.03	0.82±0.05	0.82±0.04	0.82±0.05	0.84±0.09	0.91±0.06	0.89±0.04	0.84±0.06

The last column shows the results derived across the whole age range.

Supplementary Table 7. Regional mean ± standard deviation (SD) of derived RD values averaged over participants within each age decade.

	Mean ± SD RD x 10^{-3} values (mm^2/s)								
	20-29 yrs.	30-39 yrs.	40-49 yrs.	50-59 yrs.	60-69 yrs.	70-79 yrs.	80-89 yrs.	90-99 yrs.	20-99 yrs.
Superior cerebellar peduncle	0.52±0.04	0.52±0.04	0.51±0.04	0.47±0.03	0.47±0.04	0.45±0.05	0.48±0.05	0.48±0.01	0.49±0.05
Middle cerebellar peduncle	1.41±0.03	1.35±0.28	1.41±0.36	1.13±0.25	1.36±0.30	1.43±0.30	1.52±0.38	1.32±0.17	1.39±0.33
Inferior cerebellar peduncle	0.57±0.05	0.57±0.05	0.56±0.05	0.55±0.07	0.56±0.06	0.55±0.08	0.58±0.07	0.57±0.08	0.56±0.06
Cerebral peduncle	0.44±0.05	0.44±0.06	0.46±0.06	0.46±0.04	0.45±0.04	0.49±0.07	0.51±0.05	0.49±0.08	0.46±0.06
Corticospinal tract	0.57±0.09	0.55±0.04	0.55±0.07	0.56±0.06	0.56±0.07	0.57±0.06	0.58±0.06	0.54±0.06	0.56±0.07
Pontine tract	0.60±0.06	0.61±0.03	0.61±0.05	0.59±0.07	0.59±0.07	0.61±0.09	0.64±0.08	0.64±0.07	0.61±0.07
Lemniscus tract	0.49±0.06	0.50±0.03	0.47±0.05	0.48±0.06	0.45±0.04	0.44±0.05	0.49±0.05	0.47±0.03	0.48±0.05
Whole white matter	0.82±0.11	0.82±0.11	0.80±0.14	0.78±0.13	0.79±0.12	0.84±0.12	0.91±0.21	0.80±0.08	0.82±0.15
Midbrain white matter	0.80±0.06	0.80±0.06	0.81±0.05	0.85±0.05	0.86±0.04	0.90±0.04	0.94±0.06	1.04±0.05	0.85±0.08
Pons white matter	0.72±0.06	0.70±0.03	0.71±0.07	0.69±0.05	0.71±0.04	0.72±0.05	0.76±0.07	0.78±0.05	0.72±0.06
Medulla white matter	1.02±0.12	0.99±0.10	0.97±0.13	1.07±0.14	1.04±0.11	1.12±0.15	1.20±0.21	1.27±0.27	1.05±0.17
Red nucleus	0.49±0.05	0.50±0.04	0.48±0.04	0.47±0.06	0.46±0.04	0.48±0.06	0.53±0.05	0.50±0.01	0.49±0.05
Subthalamic nucleus	0.78±0.10	0.76±0.12	0.78±0.10	0.90±0.11	0.91±0.07	1.07±0.17	1.21±0.20	1.29±0.07	0.90±0.20
Substantia nigra	0.60±0.05	0.60±0.04	0.60±0.05	0.61±0.04	0.61±0.05	0.62±0.08	0.70±0.06	0.66±0.06	0.62±0.07

The last column shows the results derived across the whole age range.
